# Fibrillar structures induced by a plant reovirus target mitochondria to activate typical apoptotic response and promote viral infection in insect vectors

**DOI:** 10.1371/journal.ppat.1007510

**Published:** 2019-01-17

**Authors:** Qian Chen, Limin Zheng, Qianzhuo Mao, Jiejie Liu, Haitao Wang, Dongsheng Jia, Hongyan Chen, Wei Wu, Taiyun Wei

**Affiliations:** 1 Fujian Province Key Laboratory of Plant Virology, Institute of Plant Virology, State Key Laboratory of Ecological Pest Control for Fujian and Taiwan Crops, Vector-borne Virus Research Center, Fujian Agriculture and Forestry University, Fuzhou, Fujian, PR China; 2 Key Laboratory of Pest Management of Horticultural Crop of Hunan Province, Hunan Plant Protection Institute, Hunan Academy of Agricultural Science, Changsha, PR China; University of Cambridge, UNITED STATES

## Abstract

Numerous plant viruses that cause significant agricultural problems are persistently transmitted by insect vectors. We wanted to see if apoptosis was involved in viral infection process in the vector. We found that a plant reovirus (rice gall dwarf virus, RGDV) induced typical apoptotic response during viral replication in the leafhopper vector and cultured vector cells, as demonstrated by mitochondrial degeneration and membrane potential decrease. Fibrillar structures formed by nonstructural protein Pns11 of RGDV targeted the outer membrane of mitochondria, likely by interaction with an apoptosis-related mitochondrial protein in virus-infected leafhopper cells or nonvector insect cells. Such association of virus-induced fibrillar structures with mitochondria clearly led to mitochondrial degeneration and membrane potential decrease, suggesting that RGDV Pns11 was the inducer of apoptotic response in insect vectors. A caspase inhibitor treatment and knockdown of caspase gene expression using RNA interference each reduced apoptosis and viral accumulation, while the knockdown of gene expression for the inhibitor of apoptosis protein improved apoptosis and viral accumulation. Thus, RGDV exploited caspase-dependent apoptotic response to promote viral infection in insect vectors. For the first time, we directly confirmed that a nonstructural protein encoded by a persistent plant virus can induce the typical apoptotic response to benefit viral transmission by insect vectors.

## Author summary

Of the approximately 700 known plant viruses, more than 75% are transmitted by insects. Numerous plant viruses can replicate inside the cells of the insects. Unlike in the plant hosts, the viruses do not seem to cause disease in the insect vectors that carry them. Here, we report that the replication of a plant reovirus, rice gall dwarf virus (RGDV), activated the apoptotic response in limited areas of leafhopper vectors during viral replication. Interestingly, fibrillar structures constituted by nonstructural protein Pns11, which is encoded by RGDV, targeted the mitochondria and induced apoptotic response in the absence of viral replication, possibly via the specific interaction of RGDV Pns11 with an apoptosis-related mitochondrial outer membrane-associated protein. Our findings further suggest that the activation of apoptotic response facilitates efficient viral infection, whereas inhibition of apoptotic response blocks viral infection in insect vectors. This work presents a novel discovery that a plant reovirus induces typical apoptotic response and thus promotes its transmission by insect vectors.

## Introduction

In mammals, viral infection can induce or activate apoptosis, a process of programmed cell death, which generally is important in the regulation of viral pathogenesis [1]. Apoptosis is a normal process during development and aging to regulate cell populations in multicellular organisms [2–3]. Caspases, a family of cysteine proteases, are crucial proteases responsible for the execution of the apoptotic cascade, while the inhibitor of apoptosis protein (IAP) serves as a pivotal regulator of apoptosis [4]. Apoptosis is triggered either via an extrinsic death receptor or an intrinsic mitochondria-dependent pathway [5–6]. The initial event of mitochondria-dependent apoptosis is the loss of mitochondrial membrane potential, leading to the release of apoptosis-related factors associated with the mitochondrial membranes [7–10]. Later, the chromatin is cleaved into nucleosomal fragments, and apoptotic bodies are generated [11]. These fundamental stages are first elucidated for mammalian systems, due to the important function of apoptosis in development and diseases [2]. Although apoptosis is commonly involved in viral pathogenesis, some viruses appear to have evolved to exploit this mechanism to promote their survival and replication in different ways [12–14]. Thus, the role of apoptosis in host–virus interactions is diverse among different viruses.

Many plant viruses that cause significant agricultural problems are transmitted via insect vectors such as thrips, aphids, leafhoppers and planthoppers in a persistent manner [15]. Growing evidence has shown that the persistent transmission of viruses causes only a limited adverse effect, rather than pathogenesis in their insect vectors [15–20]. We now know that a conserved small interfering RNA (siRNA) antiviral response is triggered by the replication of viruses in the insect vectors to modulate a metastable balance between viral accumulation and adverse effects, allowing for viral persistence and highly efficient spread in nature [15, 21–24]. Generally, persistent infection by arthropod-borne viruses (arboviruses) can induce apoptosis in mosquito and *Drosophila* vectors, but it is usually restricted to a low level to avoid serious damage to the insects [14, 25–27]. The apoptosis induced by arboviruses may serve as an innate antiviral mechanism to protect against or benefit viral transmission by insect vectors [12, 14]. The cytopathologic changes caused by virus-induced apoptosis may also damage functionally relevant tissues and organs, decreasing insect fitness. Using the terminal deoxynucleotidyl transferase dUTP nick-end labeling (TUNEL) assay, Huang et al. demonstrated that apoptotic signs appeared in limited regions of the principal salivary glands in brown planthopper infected with rice ragged stunt virus, a plant reovirus [28]. However, the functional roles for apoptosis induced by persistent plant viruses in insect vectors are still poorly understood. This gap in knowledge is, in part, attributable to the lack of reliable tools such as insect vector cell cultures for real-time analysis of virus-induced apoptosis in insect vector cells.

In the present study, we used continuous cell cultures derived from insect vectors to trace the apoptosis process induced by rice gall dwarf virus (RGDV), a plant reovirus that was first described in 1979 in Thailand and caused a severe disease of rice in southern China and Southeast Asia [29]. RGDV is mainly transmitted with high efficiency in a persistent-propagative manner by the leafhopper vector *Recilia dorsalis* (Hemiptera: Cicadellidae) [17]. RGDV has icosahedral and double-shelled particles approximately 65 to 70 nm in diameter [24]. Its genome consists of 12 double-stranded RNA (dsRNA) segments, encoding six structural proteins and six nonstructural proteins [29]. The outer capsid shell is composed of the major outer capsid protein P8 and the minor outer capsid protein P2 [24]. Nonstructural protein Pns11 assembles into fibrillar or tubular structures to facilitate viral spread in insect vectors [17, 30–31]. The leafhopper *R*. *dorsalis* and its cultured cells support the efficient propagation of RGDV to a high titer in a persistent and nonlethal infection that causes limited damage [17, 19]. We have demonstrated that the siRNA antiviral pathway modulates the persistent infection of RGDV in *R*. *dorsalis*, thus preventing serious harm [24].

However, we still do not know how RGDV regulates physiological processes in *R*. *dorsalis* to permit effective viral propagation. Previously, we found that RGDV infection could directly remodel and utilize a variety of cellular structures and pathways for efficient propagation in its insect vectors [30–32]. For example, RGDV particles were often observed to associate with the bundles of fibrillar structures to facilitate viral infection [30–31]. Furthermore, RGDV particles are distributed close to the periphery of degenerated mitochondria during viral replication of vector cells, suggesting that mitochondria might support the energy demands of viral propagation [33]. The degeneration of virus-associated mitochondria surprised us, suggesting that RGDV infection may induce apoptotic response in insect vector cells and adversely affect the insects. Here, by applying a system that combines insect vector cell cultures, immunofluorescence and electron microscopy, we revealed that the fibrillar structures that are composed of nonstructural protein Pns11 of RGDV, targeted mitochondria and activated typical apoptotic response to promote viral infection and transmission by insect vectors.

## Results

### RGDV infection induces apoptotic response in insect vector cells

**Fig 1:**
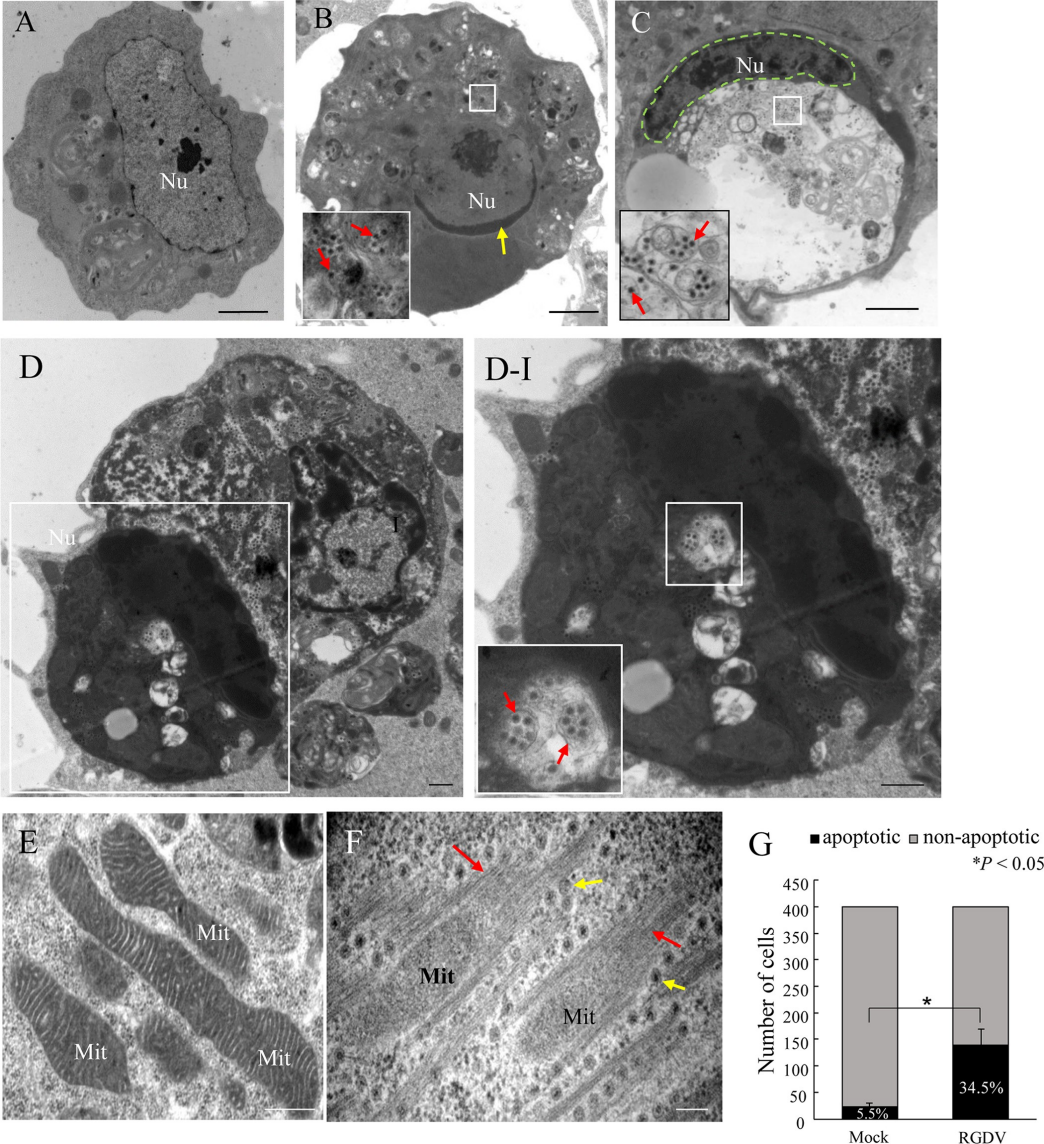
RGDV infection induced morphological hallmarks of apoptosis in continuous cultured cells of *R*. *dorsalis*. Cell cultures derived from *R*. *dorsalis* were originally established from the embryonic fragments dissected from eggs and subcultured for more than 100 generations. Cultured cells were inoculated with purified RGDV (MOI of 1) and processed at 48 hpi for TEM. The infection rate at this time was approximately 70%. (A) Mock-infected cultured cells of *R*. *dorsalis* with normal cellular morphology. (B) Condensed chromatin in cells infected by RGDV particles (red arrows) was marginalized along the nuclear inner envelope and appeared as cup-shaped masses (yellow arrow). (C) The nucleus (green dotted line) in cells infected by RGDV particles (red arrows) was crescent-shaped. (D) Large cell engulfing an apoptotic body containing RGDV particles (red arrows). (E) Intact mitochondria with distinct, tightly involute cristae in mock-infected cells. (F) Degenerated mitochondria with indistinct, diffuse cristae surrounded by fibrillar structures (red arrows) in virus-infected cultured cells of *R*. *dorsalis*. Yellow arrows indicate RGDV particles along the edges of fibrillar structures. Inserts in panels B, C and D-I are enlargements of the respective boxed areas. Panel D-I is an enlargement of the boxed area in panel D. Nu, nucleus; Mit, mitochondria. Bars, 2 μm (A-C), 500 nm (D, E), 100 nm (F). (G) Number of apoptotic cells in RGDV-infected cells (RGDV) was significantly higher than in mock-infected cells. His-Mg buffer-treated cells served as mock controls. Mean (±standard deviation [SD]) from three biological replicates are shown. *P < 0.05. Data were analyzed with a two-tailed *t*-test in GraphPad Prism 7.

RGDV exerts an adverse effect on its vector *R*. *dorsalis*, including reduced survival, emergence, fecundity and longevity of adults [19]. To explore how the virus causes these adverse effects, we first investigated whether RGDV infection caused apoptotic changes in continuous cultured cells of *R*. *dorsalis*, which were originally established from embryonic fragments dissected from eggs [17]. At 72 h post-inoculation (hpi) with RGDV at a multiplicity of infection (MOI) of 1, bright field microscopy showed that the infected cultured insect cells had slight cytopathological changes, such as cell clumping and loss of confluent monolayers (S1 Fig). Electron microscopy showed that RGDV-infected cells had apoptotic characteristics, including crescent-shaped nuclei and condensed and marginalized chromatin, compared with the round nuclei and finely dispersed chromatin in mock-infected cells (Fig 1A–1C). At this time, virus-containing apoptotic bodies, the typical characteristic of the end stage of apoptosis were present (Fig 1D). The mitochondria in RGDV-infected cells appeared to be degenerating, and cristae were diffuse and indistinct (Fig 1E and 1F). Bundles of fibrillar structures, absent in virus-free cells, were in contact with the periphery of these degenerated mitochondria (Fig 1F). Some RGDV particles were closely associated with the free ends of the virus-induced fibrillar structures and along their edges (Fig 1F). Thus, RGDV infection caused cytopathological changes that included the hallmarks of apoptosis. To better understand the prevalence of RGDV-induced apoptotic response in cultured cells, we examined 400 cells in mock- or RGDV-infected treatment to count the number of apoptotic cells using electron microscopy and found that 34.5% of the cells were apoptotic, significantly higher than in the mock treatment (Fig 1G). Therefore, RGDV specifically induced specific morphology changes of apoptosis in cultured cells of *R*. *dorsalis*.

**Fig 2:**
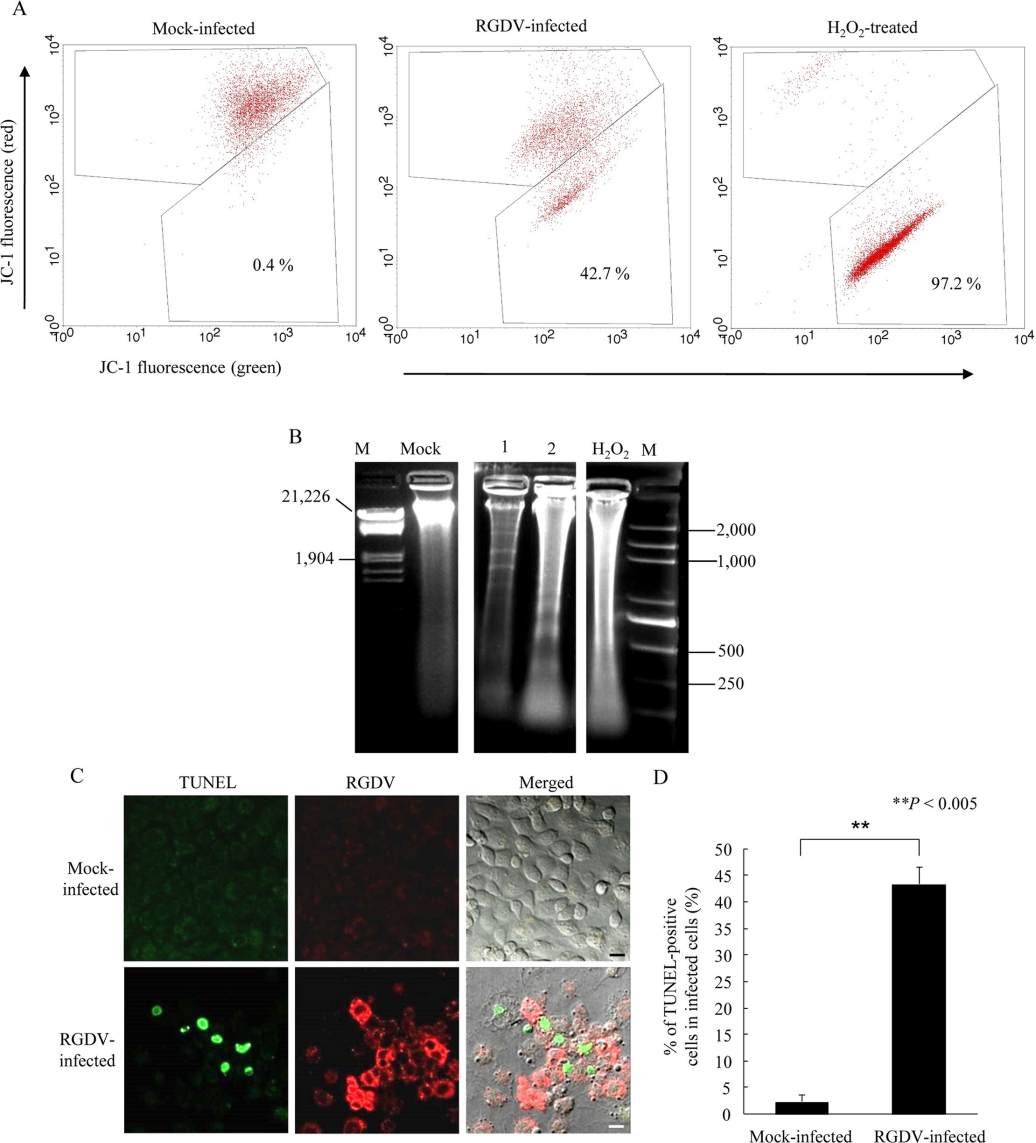
RGDV infection triggered the apoptotic response in continuous cultured cells of *R*. *dorsalis*. (A) Mitochondrial membrane potential decreased in RGDV-infected cells at 48 hpi as determined by flow cytometric analysis. Loss of mitochondrial membrane potential is displayed as the change in JC-1 fluorescence from red (JC-1 aggregates) to green (JC-1 monomers). Mock-infected, RGDV-infected and H_2_O_2_-treated cells were loaded with JC-1. Each panel and blot are representative of three separate experiments. (B) Chromosomal DNA of cultured cells was fragmented into nucleosomal oligomers as shown by gel electrophoresis. Lane M, DNA marker; lanes 1 and 2, DNA extracts from virus-infected cultured cells at 72 hpi. (C) Chromosomal fragmentation in RGDV-infected cells was TUNEL-positive. At 72 hpi, *R*. *dorsalis* cells growing in a monolayer were *in situ* labeled with TUNEL (green) and immunolabeled with virus-rhodamine (red), then examined by confocal microscopy. Mock-infected cells served as a control. Bars, 10 μm. (D) Percentage of TUNEL-positive cells in RGDV- or mock-infected cells at 72 hpi. His-Mg buffer-treated cells served as the mock controls. Means (±SD) from three biological replicates are shown. **P < 0.005. Data were analyzed with a two-tailed *t*-test in GraphPad Prism 7.

One of the key characteristics of apoptosis at an early stage is the disruption of the mitochondrial membrane potential [2, 34]. The degeneration of mitochondria in RGDV-infected cells is probably caused by a change of the mitochondrial membrane potential, which initiates a mitochondria-dependent apoptotic cascade. We thus used the JC-1 assay, a flow cytometry-based method widely used to detect any changes in mitochondrial membrane potential. Continuous cultured cells of *R*. *dorsalis* were inoculated with RGDV at a MOI of 1. At 48 hpi, RGDV infection caused the mitochondrial membrane potential to decrease in 42.7% of the cultured leafhopper cells, which was significantly higher than in the mock-infected treatment (Fig 2A). Thus, RGDV infection induced early-stage apoptotic response. At 72 hpi, the infected cells were then examined for nucleosomal fragmentation, a hallmark event of later-stage apoptosis [11]. At this later time, a clear ladder of DNA fragments was detected in RGDV-infected cells, but not in the mock, which had a single intact chromosomal DNA band (Fig 2B). Furthermore, the TUNEL assay, widely used to detect apoptotic bodies [35], showed positive apoptotic signals in virus-infected regions, but none in the uninfected cells (Fig 2C). We calculated that about 43% of infected cells were TUNEL-positive (Fig 2D). These results strongly indicated that RGDV infection specifically induced the early and later events of apoptotic response in insect vector cells.

### Nonstructural protein Pns11 of RGDV targets and induces mitochondrial degeneration

**Fig 3:**
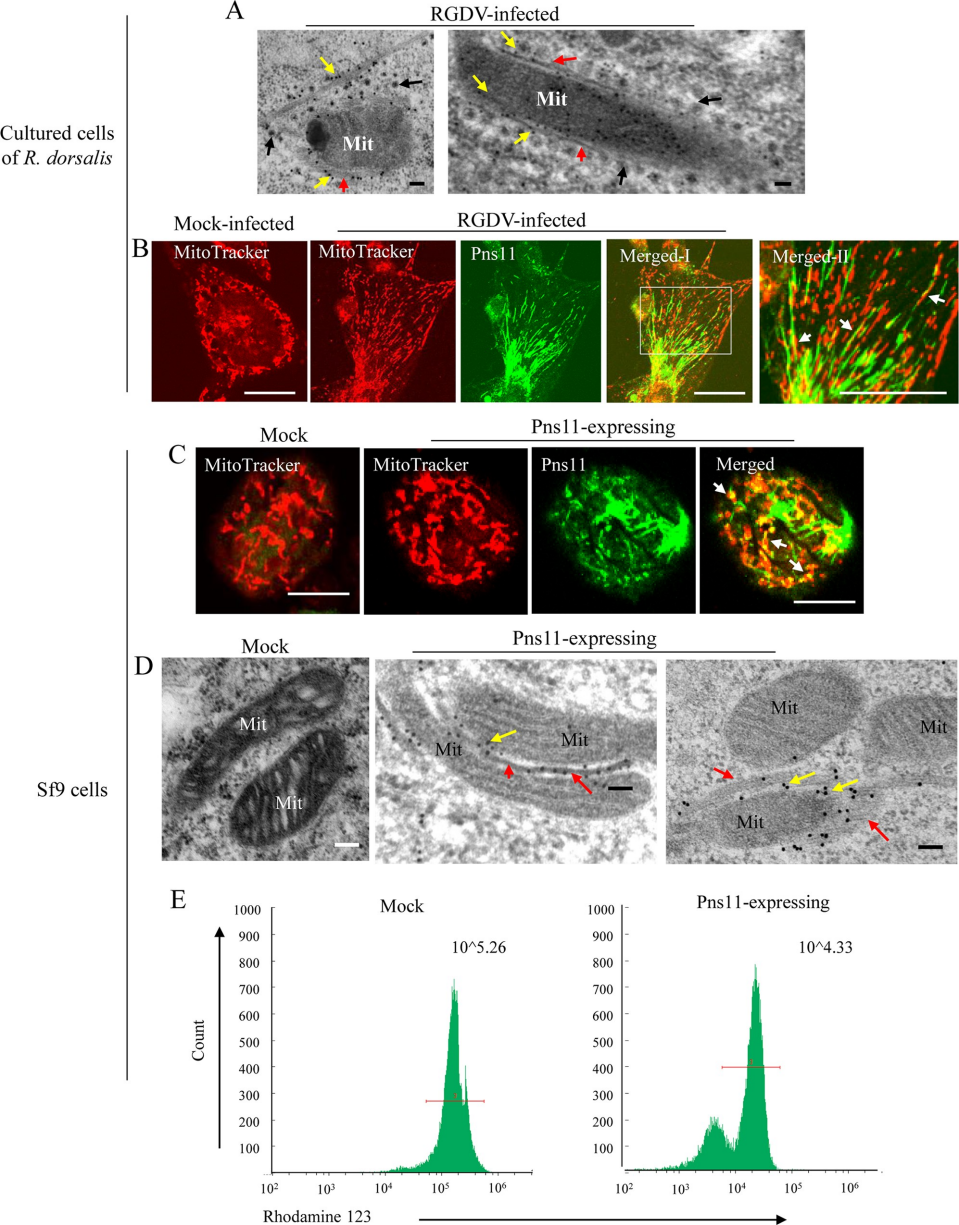
Fibrillar structures composed of RGDV Pns11 were associated with mitochondria by interacting with VDAC. (A) Immunogold labeling of Pns11 on fibrillar structures (red arrows) at the periphery of degenerated mitochondria. Yellow arrows mark gold particles. Black arrows mark viral particles. Bar, 100 nm. (B) Immunofluorescence assay showing colocalization of Pns11-specific fibrillar structures with mitochondria (arrows) in virus-infected *R*. *dorsalis* cells at 48 hpi. Mock- or virus-infected cells were immunostained with Pns11-FITC (green) and MitoTracker (red). Panel Merged-II is an enlargement of boxed area in panel Merged-I. His-Mg buffer-treated cells served as mock controls. Bars, 10 μm. (C) Immunofluorescence assay showing colocalization of RGDV Pns11 with mitochondria (arrows) in Pns11-expressing Sf9 cells at 48 hpi. Mock or Pns11-expressing Sf9 cells were immunostained with Pns11-FITC (green) and MitoTracker (red). Bars, 10 μm. (D) Immunogold labeling of Pns11 in fibrillar structures (red arrows) surrounding mitochondria with diffuse cristae in Sf9 cells at 48 hpi. Yellow arrows mark gold particles. Bars, 100 nm. (E) Mitochondrial membrane potential was reduced in Pns11-expressing Sf9 cells at 72 hpi as determined by flow cytometric analysis. Mock or Pns11-expressing Sf9 cells were stained with rhodamine 123. Sf9 cells inoculated with empty baculovirus vector served as a negative control. Mit, mitochondria. Each panel is representative of three independent biological experiments.

Because RGDV infection of insect vector cells has been shown to induce the formation of various inclusions composed of nonstructural proteins for viral replication or spread [29–31, 36], we used immunoelectron microscopy to investigate which viral nonstructural proteins (Pns4, Pns7, Pns9, Pns10, Pns11 or Pns12) were involved in the formation of virus-associated fibrillar structures along the degenerated mitochondria. Immunoelectron microscopy showed that Pns11-specific IgG specifically recognized the fibrillar structures surrounding the degenerated mitochondria (Fig 3A). Confocal microscopy further confirmed that some Pns11-specific fibrillar structures colocalized with the mitochondria, which were stained by MitoTracker Red in virus-infected *R*. *dorsalis* cells (Fig 3B). Thus, the fibrillar structures composed of Pns11 of RGDV apparently targeted the mitochondria and may induce mitochondrial degeneration during viral infection of insect vector cells.

Previously, we showed that expression of RGDV Pns11 alone can induce the formation of fibrillar or tubule-like structures in cells of the nonhost *Spodoptera frugiperda* (Sf9) [17]. To determine whether Pns11 of RGDV had an inherent ability to target and induce mitochondrial degeneration, Sf9 cells were inoculated with recombinant baculovirus that expressed Pns11. Confocal microscopy demonstrated that some Pns11-specific fibrillar structures colocalized with the mitochondria stained by MitoTracker Red in the cytoplasm of Sf9 cells at 48 hpi (Fig 3C). Immunoelectron microscopy further showed that the fibrillar structures-associated mitochondria were degenerating, with diffuse and indistinct cristae in the cytoplasm of Sf9 cells at 48 hpi (Fig 3D). These results illustrated that RGDV Pns11 had an inherent ability to target and induce mitochondrial degeneration in the absence of viral infection.

To characterize the cytopathological effect of Pns11 of RGDV in Sf9 cells, we stained cells with trypan blue to observe the cellular phenotypes and measure cell viability using a cell counter. Bright field microscopy showed that the viability of Sf9 cells had greatly decreased after Pns11 accumulation at 72 hpi, indicating the relative cytotoxicity of Pns11 (S2A and S2B Fig). We then used rhodamine 123, a specific fluorescent dye used to assess mitochondrial membrane potential [37], to determine whether Pns11 alone induces the collapse of mitochondrial membrane potential. We found that Pns11 caused an 87.1% decrease in total rhodamine 123 fluorescence intensity of Sf9 cells at 72 hpi, compared with that of the mock (Fig 3E), suggesting that Pns11 alone potentially reduced mitochondrial membrane potential. Because the disruption of the mitochondrial membrane potential can lead to cytochrome c release [7–10], we then determined whether the cytochrome c could translocate from the mitochondrial to cytosol. The immunofluorescence assay demonstrated that at 72 hpi, the cytochrome c in Pns11-expression Sf9 cells was largely localized within the cytosol, but not together with the mitochondria (S2C Fig). Thus, Pns11 alone potentially induced the release of apoptosis-related factors and the subsequent apoptotic cascade.

**Fig 4:**
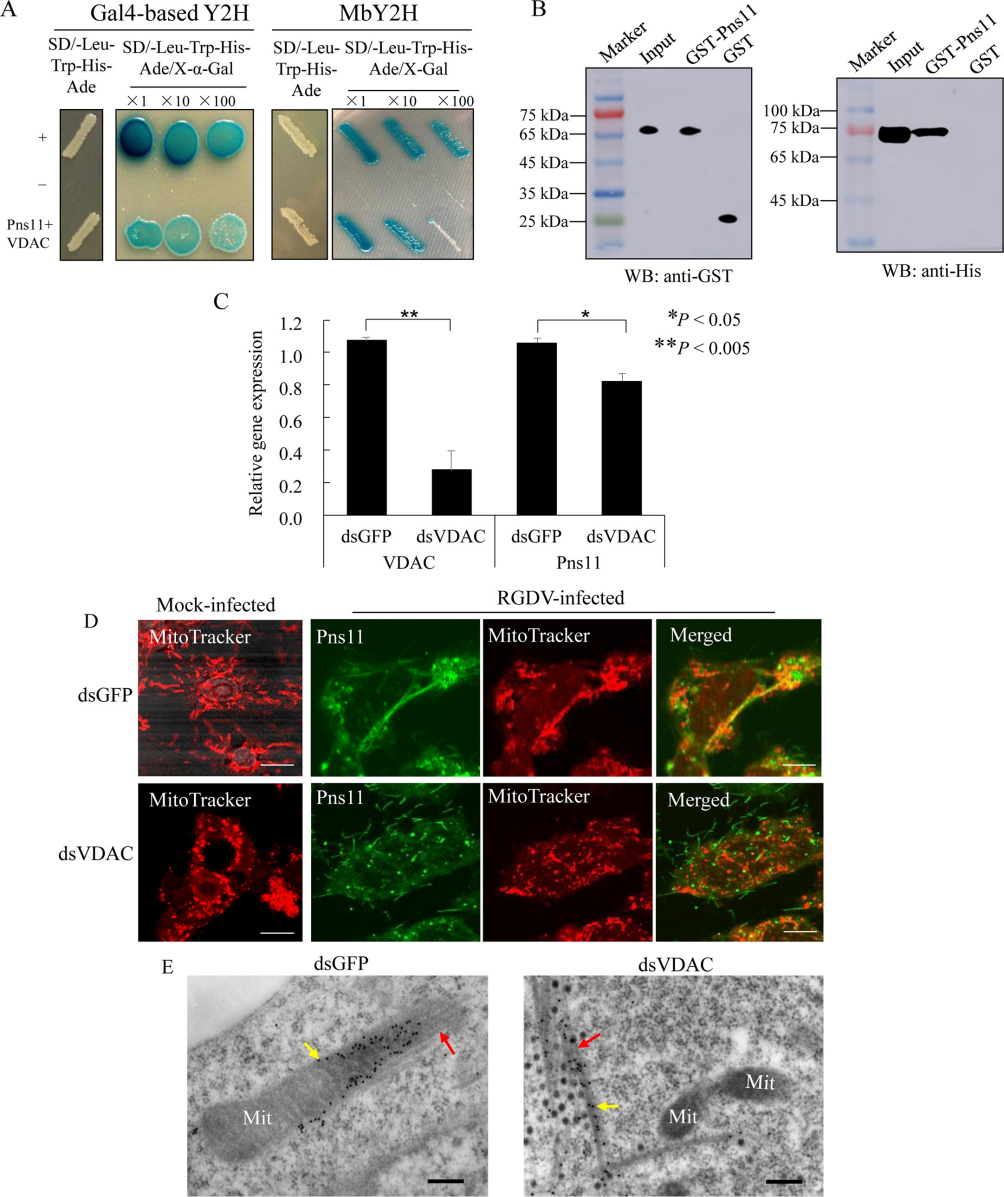
Pns11 of RGDV specifically interacted with VDAC on the outer membrane of mitochondria. (A) RGDV Pns11 specifically interacted with VDAC in Y2H assays. For the Gal-4 based Y2H assay, transformants on plate of SD/Trp-Leu-His-Ade medium are labeled as follows: +, Positive control, i.e., pGBKT7-53/ pGADT7-T;–, negative control, i.e., pGBKT7-Lam/pGADT7-T; Pns11+VDAC, pGBKT7-Pns11/pGADT7-VDAC. Serially diluted yeast cultures appeared blue in the β-galactosidase assay. For the DUALmembrane Y2H assay, transformants on plate of SD/Trp-Leu-His-Ade medium are labeled as follows: +, positive control (pTSU2-APP/pNubG-Fe65);–, negative control (pTSU2-APP/pPR3-N); Pns11+VDAC, pBT-STE-Pns11/ pBT3-N-VDAC. Serially diluted yeast cultures appeared blue in the β-galactosidase assay. (B) GST pull-down assay demonstrated the interaction of Pns11 with VDAC. Protein GST and recombinant protein GST-Pns11 were respectively incubated with cell lysate expressing protein His-VDAC. Pull-down products were detected using Western blotting. An antibody against GST was used to detect GST and GST-Pns11; an antibody against His was used to detect bound proteins. (C) RT-qPCR assay of relative expression of VDAC and RGDV Pns11 genes in *R*. *dorsalis* cells after dsRNA treatment. The dsRNA-treated cells were inoculated with purified RGDV (MOI of 0.1), then harvested at 48 hpi. Means (±SD) from three biological replicates are shown. *P < 0.05, **P < 0.005. Data were analyzed with a two-tailed *t*-test in GraphPad Prism 7. (D) Immunofluorescence assay showing that knockdown of VDAC expression decreased the number of Pns11 fibrillar structures at 48 hpi. Mock- or RGDV-infected cells after treatment with dsGFP or dsVDAC were immunostained with Pns11-FITC (green) and MitoTracker (red). His-Mg buffer-treated cells served as mock controls. Bars, 10 μm. (E) Immunogold labeling of Pns11 on fibrillar structures (red arrows) in dsGFP- or dsVDAC-treated infected cultured cells of *R*. *dorsalis* at 48 hpi. Yellow arrows mark gold particles. Mit, mitochondria. Bar, 200 nm.

A yeast two-hybrid (Y2H) assay was then used to screen a cDNA library of *R*. *dorsalis* to identify putative mitochondrial factors interacting with RGDV Pns11. From this library screen, 116 colonies of 207 positive ones were randomly sequenced. Finally, 36 sequences were annotated using the BLASTX program in GenBank. Among these candidates, an apoptosis-related protein named voltage-dependent anion channel (VDAC) (also called mitochondrial porin) captured our attention. The VDAC, a class of porin channel located on the outer mitochondrial membrane, serves as a major diffusion pathway for ions and metabolites [38]. This protein plays a crucial role in apoptosis [39]. At the early stage of apoptosis, the VDAC increases the permeability of mitochondrial membrane to allow the release of apoptotic factors, such as cytochrome c and apoptosis-inducing factor, then initiates the apoptotic cascade [39]. Therefore, the VDAC of *R*. *dorsalis* was analyzed further. Based on the transcriptome data from *R*. *dorsalis* in our lab, the BLASTX searching method in the GenBank demonstrated the 90% similarity of putative full-length open reading frame (ORF) of VDAC (846 bp long) with counterpart of *Homalodisca vitripennis* (S3A Fig). Then this putative ORF of VDAC was amplified, and the gene sequence was deposited in GenBank with accession number MG241500. The predicted protein product (282 amino acid residues) possessed characteristic porin3 domains (S3B Fig), which can form a β-barrel to span the mitochondrial outer membrane [40], but did not show the significant predicted transmembrane domains analyzed with TMHMM. The phylogenic analysis revealed that the amino acid sequence of VDAC clustered with those of other insect species in the order Hemiptera (S3A Fig). Both Gal4 transcriptional activator-based and membrane-based yeast two-hybrid (MbY2H) systems were applied and revealed the strong interaction between VDAC and Pns11 (Fig 4A). We then used a glutathione *S*-transferase (GST) pull-down assay to confirm such interaction. The GST-tag and His-tag were fused to the N-terminal of Pns11 and VDAC, respectively, to express fusion proteins GST-Pns11 and His-VDAC. The result showed that the purified GST-Pns11 pulled down His-VDAC from cell lysates (Fig 4B). By contrast, no such interaction was obtained with the purified GST (Fig 4B). Thus, RGDV Pns11 specifically interacted with the VDAC, suggesting that the VDAC was likely the target protein mediating the binding of Pns11-specific fibrillar structures with the mitochondria.

To confirm this possibility, we then performed RNA interference (RNAi) experiments to test the effect of the reduced expression of VDAC gene on the interaction between Pns11 and VDAC. Cultured cells of *R*. *dorsalis* were transfected with synthesized dsRNA targeting the gene of VDAC (dsVDAC), then infected with purified RGDV (MOI of 1). RT-qPCR assay demonstrated that dsVDAC treatment caused approximately 70% or 20% reduction in the relative expression of VDAC or Pns11 at 48 hpi, respectively (Fig 4C), suggesting that the formation of Pns11-specific fibrillar structures was independent of VDAC. Immunofluorescence and immunoelectron microscopy further demonstrated that at 48 hpi, dsVDAC treatment significantly inhibited the colocalization of Pns11-specific fibrillar structures with the mitochondria in cultured cells of *R*. *dorsalis* (Fig 4D and 4E). Taken together, our results indicated that the target of Pns11-specific fibrillar structures with the mitochondria may depend on the specific interaction of RGDV Pns11 with an apoptosis-related mitochondrial outer membrane protein.

### Caspase-dependent apoptotic response promotes viral infection in insect vector cells

**Fig 5:**
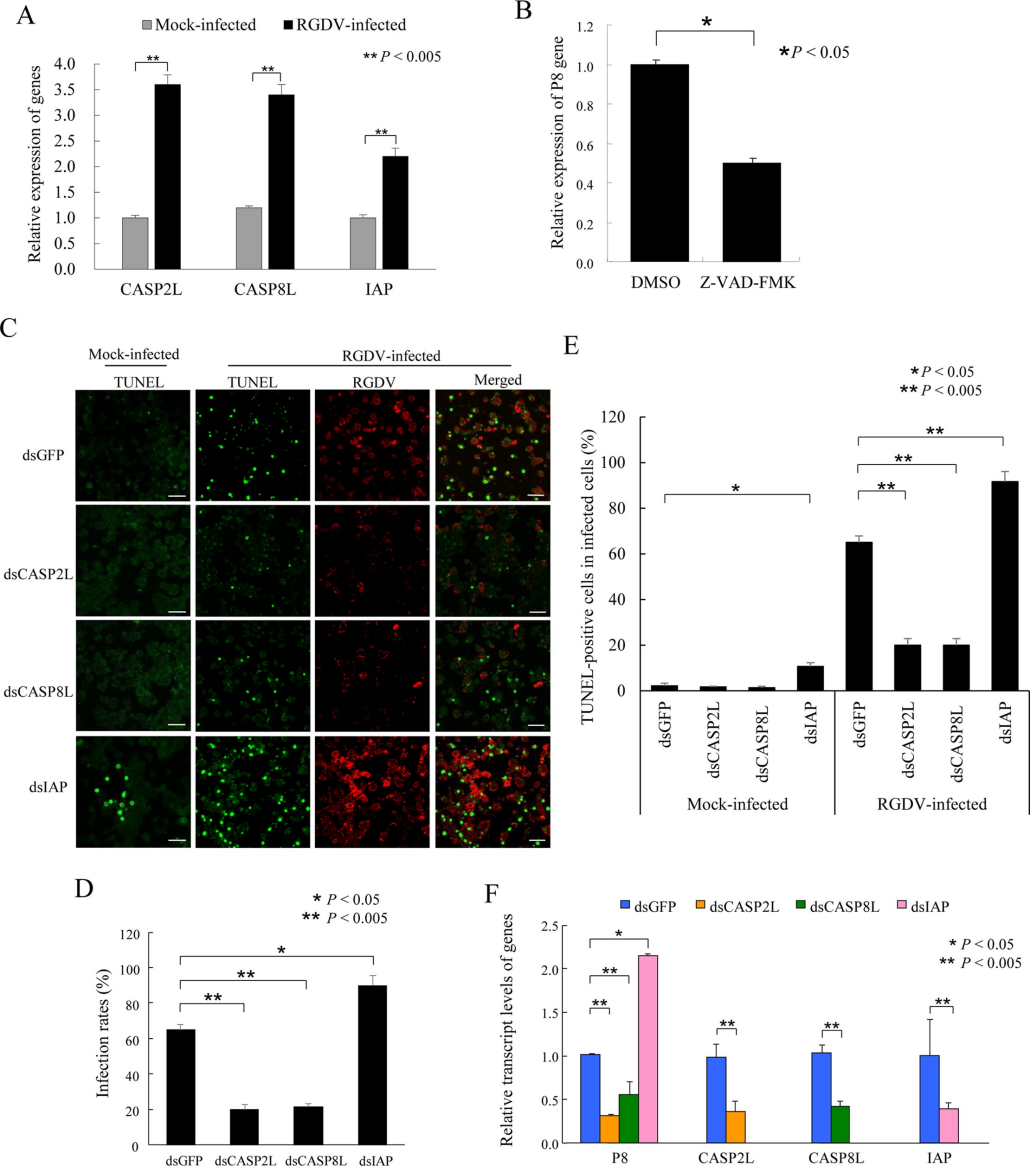
The apoptotic response induced by RGDV infection occurred via a caspase-dependent pathway in continuous cultured cells of *R*. *dorsalis*. (A) RT-qPCR assay showing the relative gene expression for CASP2L, CASP8L or IAP was up-regulated by RGDV infection. Cultured cells were inoculated with purified RGDV (MOI of 1), then harvested at 48 hpi. Relative gene expression of CASP2L, CASP8L or IAP at 48 hpi was normalized to the mock-infected cells. His-Mg buffer-treated cells served as mock controls. (B) RT-qPCR assay showing the relative gene expression for RGDV P8 after treatment with the pancaspase inhibitor Z-VAD-FMK. Cultured cells were incubated for 4 h with 25 μM pancaspase inhibitor Z-VAD-FMK dissolved in DMSO. DMSO treatment served as the control. Cells were inoculated with purified virus (MOI of 0.1), achieving an early infection rate of about 20–30%, then assayed by RT-qPCR assay at 48 hpi. (C) The apoptotic response induced by RGDV infection facilitated viral accumulation in insect vector cells. Cultured cells in a monolayer were treated with dsCASP2L, dsCASP8L, dsIAP or dsGFP, then inoculated with RGDV (MOI of 0.1). At 72 hpi, cells were *in situ* labeled with TUNEL (green) and immunolabeled with virus-rhodamine (red), then examined by confocal microscopy. The TUNEL assay for dsRNAs-treated and His-Mg buffer-treated cells served as mock controls. Images are representative of three independent experiments. Bars, 40 μm. (D, E) The percentage of viral infection (D) or TUNEL-positive cells (E) in RGDV- or mock-infected cells treated with dsRNAs at 72 hpi. His-Mg buffer-treated cells served as mock controls. (F) RT-qPCR assay showing the relative gene expression for RGDV P8, CASP2L, CASP8L, and IAP after respective dsRNA treatment. The dsRNA-treated cells were inoculated with purified RGDV (MOI of 0.1), then assayed or harvested at 48 hpi. Means (±SD) from three independent biological replicates are shown. *P < 0.05, **P < 0.005. Data were analyzed with a two-tailed *t*-test in GraphPad Prism 7.

For clarifying that the apoptotic pathway is induced by RGDV infection, IAP and two caspase orthologs, caspase-2-like (CASP2L) and caspase-8-like (CASP8L), were first identified in transcriptome data from *R*. *dorsalis*. The full-length ORFs of CASP2L, CASP8L and IAP genes of *R*. *dorsalis* were amplified, and each gene sequence was deposited in GenBank (accessions MG241499, MG241497 and MG241498, respectively). Phylogenic analysis showed that the amino acid sequences of CASP2L and CASP8L clustered with those of other insect species (S4 Fig). To determine the expression profiles of apoptosis-related genes during viral infection, cultured cells of *R*. *dorsalis* were infected with purified RGDV (MOI of 1). At 48 hpi, an RT-qPCR assay showed that the expression of three apoptosis-related genes was increased significantly (Fig 5A). We further determined that the treatment with the broad-spectrum caspase inhibitor Z-VAD-FMK also significantly inhibited relative gene expression of RGDV P8 at 48 hpi, but did not significantly affect viability of *R*. *dorsalis* cells (Fig 5B). Thus, the typical apoptotic response induced by RGDV infection was caspase-dependent.

To confirm this result, we also silenced gene expression for CASP2L, CASP8L or IAP by RNAi to inhibit or induce apoptotic responses, respectively. Continuous cultured cells of *R*. *dorsalis* were treated with dsRNAs targeting the CASP2L, CASP8L or IAP genes or the gene for green fluorescence protein (GFP) (dsCASP2L, dsCASP8L, dsIAP or dsGFP). At 8 h post transfection, cells were inoculated with RGDV at a MOI of 0.1. At this low MOI, the early viral infection rate was low (about 20–30%), and the spread of viruses among *R*. *dorsalis* cells could be easily monitored. We also tested the efficiency of the knockdown of the targeted genes 48 h post-transfection with dsRNAs in continuous cultured cells (S5A Fig). By 48 hpi, after examining at least 1000 cells, immunofluorescence microscopy indicated that treatment with dsCASP2L or dsCASP8L decreased the percentage of infected cells from an average of 65% to 20%, compared with the dsGFP-treated cells control, respectively (Fig 5C and 5D). In contrast, treatment with dsIAP increased the percentage of infected cells from an average of 65% to 90%, compared with dsGFP-treated cells (Fig 5C and 5D). As expected, the number of TUNEL-positive cells was positively correlated with viral infection (Fig 5C–5E). Meanwhile, in dsCASP2L-, dsCASP8L- or dsGFP-treated uninfected cells, no specific TUNEL signals appeared (Fig 5C). However, about 10% of cells in the dsIAP-treated uninfected cells were TUNEL-positive, significantly lower than in the dsIAP-treated infected cells (Fig 5C). RT-qPCR assay demonstrated an approximately 70% reduction in the relative expression of CASP2L, CASP8L or IAP after treatments with dsCASP2L, dsCASP8L or dsIAP, respectively, at 48 hpi (Fig 5F), indicating that transfection with the dsRNA specific for these genes indeed triggered RNAi in *R*. *dorsalis* cells. RT-qPCR assay showed that the treatment with dsIAP increased gene expression of P8 by more than 2-fold (Fig 5F). By contrast, gene expression of P8 was reduced by the treatment with dsCASP2L or dsCASP8L by about 3- or 2-fold, respectively (Fig 5F).

**Fig 6:**
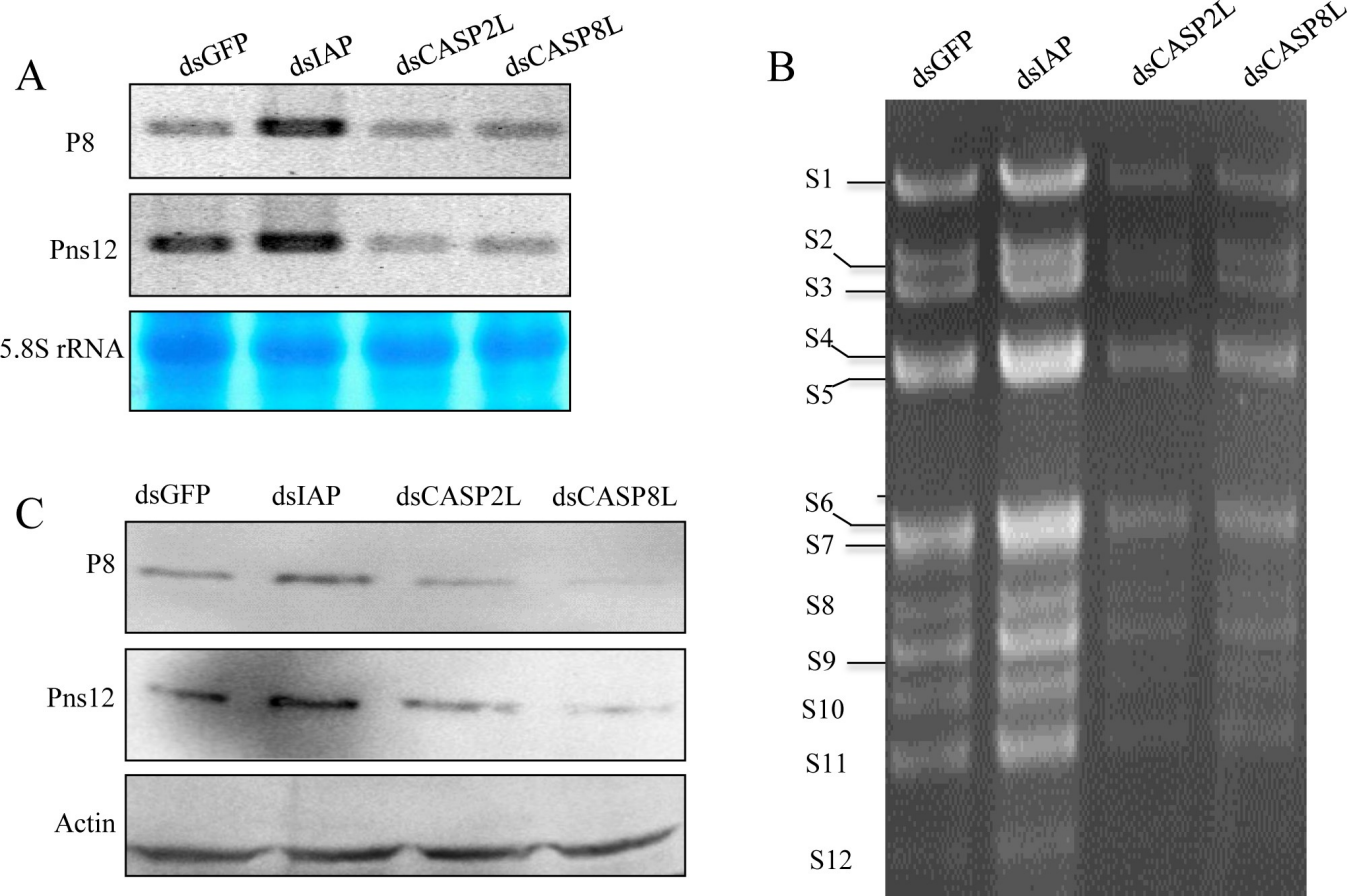
The apoptotic response induced by RGDV infection facilitated viral infection in continuous cultured cells of *R*. *dorsalis*. Effects of synthesized dsRNAs on the synthesis of viral mRNAs (A), the synthesis of viral genome dsRNAs (B), and the accumulation of viral proteins (C) in virus-infected cells. The dsRNA-treated cells were inoculated with purified RGDV (MOI of 0.1), then harvested at 72 hpi for northern blots, dsRNA isolation or western blots. (A) Approximately 5 μg of total RNAs extracted from cultured cells at 72 hpi were detected with DIG-labeled probes for viral genes of major outer capsid protein P8 or viral replication protein Pns12. Lower panel: methylene blue detection of 5.8S rRNA as a control to confirm loading of equal amounts of RNA in each lane. Image is representative of multiple experiments with multiple preparations. (B) Viral genome dsRNAs from infected cultured cells was analyzed in 1.0% agarose gel electrophoresis and ethidium bromide staining. The size classes of viral dsRNA segments are indicated. (C) Viral proteins prepared from cells lysates were analyzed at 72 hpi using polyclonal antibodies against P8 or Pns12. An actin-specific antibody was used for the control.

Northern blot analysis showed that the treatment of dsCASP2L and dsCASP8L resulted in a marked reduction of the synthesis of viral mRNAs, but the treatment of dsIAP increased the synthesis of viral mRNAs at 72 hpi (Fig 6A). Expectedly, the synthesis of viral genome dsRNAs and the accumulation of viral proteins were also decreased by treatment with dsCASP2L or dsCASP8L, but were increased by treatment with dsIAP (Fig 6B and 6C). The pattern of genomic dsRNA segments separated from purified RGDV virons is considered as the reference [41]. Thus, the apoptotic response induced by RGDV infection was beneficial for viral infection. In addition, dsIAP treatment of virus-free cells induced a low level of apoptotic response, while dsIAP treatment under viral infection significantly induced distinct apoptotic response. Totally, our results confirmed that RGDV infection inherently activated an apoptotic response in its insect vector cells.

### RGDV infection induces apoptotic response in intact insect vectors

**Fig 7:**
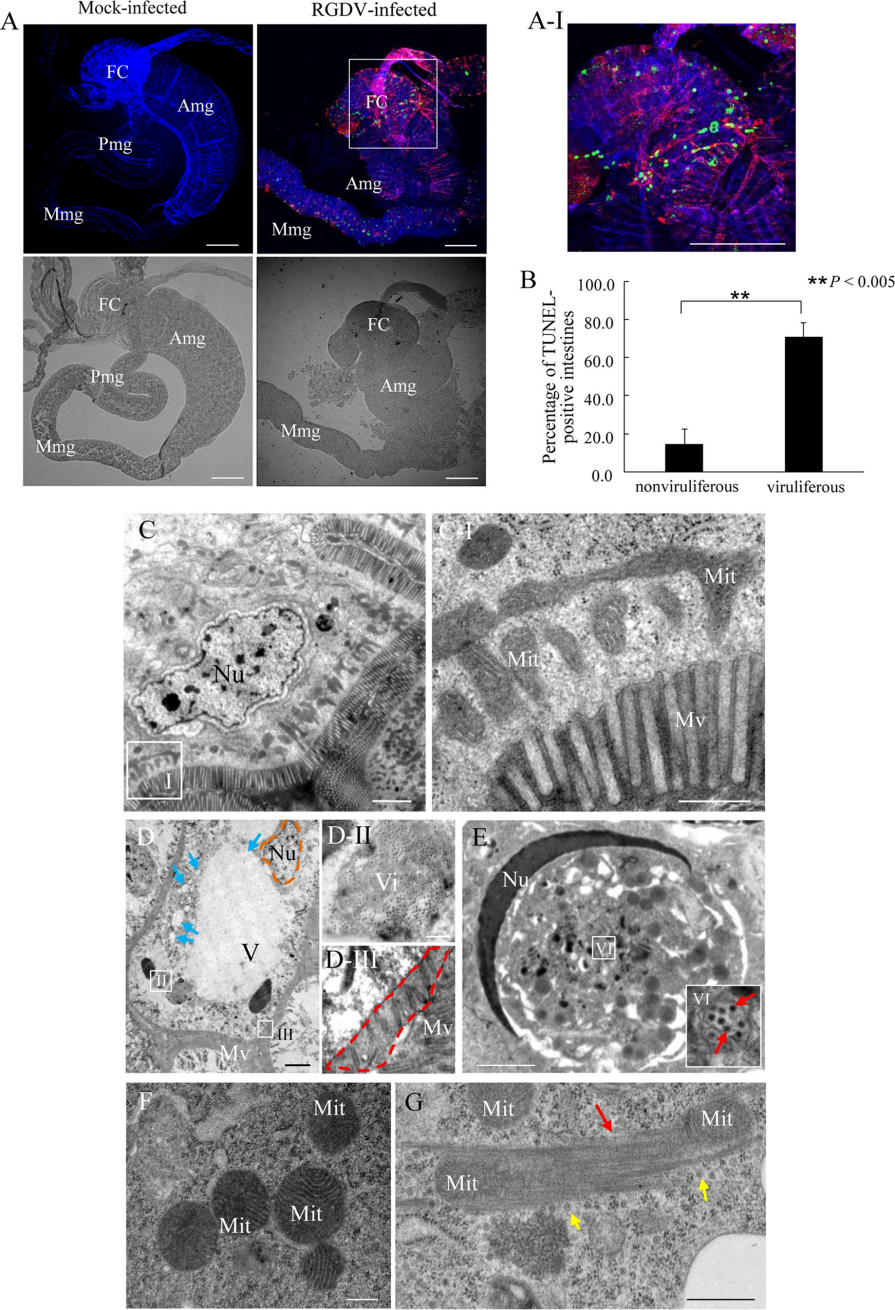
RGDV infection triggered the apoptotic response in *R*. *dorsalis*. (A) RGDV infection induced the apoptotic response in the intestines of viruliferous leafhoppers. Leafhopper intestines at 6 days padp were stained with TUNEL (green), virus-rhodamine (red) and actin dye phalloidin-Alexa Fluor 647 carboxylic acid (blue), and then examined by confocal microscopy. Panel A-I is an enlargement of the boxed area in panel A. Bright field images of the intestines are shown below the fluorescent ones. Images are representative of three independent experiments with a total 30 leafhoppers analyzed. (B) Percentage of TUNEL-positive intestines from viruliferous or nonviruliferous insects at 10 days padp. Means (±SD) from three independent biological replicates are shown. **P < 0.005. Data were analyzed with a two-tailed *t*-test in GraphPad Prism 7. (C) Intestinal epithelium in nonviruliferous leafhoppers showing normal nucleus, intact mitochondria and orderly microvilli. (D) RGDV-infected intestinal epithelium with cytopathological morphology, including nucleus shrinkage (brown dotted line), cytoplasmic vacuolation (blue arrows), and microvilli loss (red dotted line). (E) Nucleus of virus-infected cells was crescent-shaped. (F) Intact mitochondria with tightly involute cristae in nonviruliferous intestinal epithelium. (G) Degenerated mitochondria with diffuse, indistinct cristae were surrounded by bundles of fibrillar structures (red arrows) in viruliferous midgut epithelium. Yellow arrows indicate vial particles. Panels I, II and III in C and D are enlarged images of the boxed areas in respective panels. Insert VI in panel E is enlargement of the boxed area VI. FC, filter chamber; Amg, anterior midgut; Mmg, middle midgut; Pmg, posterior midgut; Nu, nucleus; Mit, mitochondrion; V, vacuolation; Mv, microvilli; Vi, virions. Bars, 100 μm (A), 2 μm (C, E), 5 μm (D), 500 nm (C-I, D-II) and 200 nm (F, G).

To determine whether RGDV infection caused apoptotic response in intact insect vector, the intestines of nonviruliferous or viruliferous *R*. *dorsalis* adults were tested using the TUNEL assay. It is known that RGDV initially infects the filter chamber epithelium of the intestines by 2 days post-first access to diseased plants (padp), then directly crosses the basal lamina into the visceral muscles at 4 days padp, from where it spreads throughout the entire intestines at 6 days padp [42]. By 10 days padp, RGDV is extensively present in the salivary glands [42]. Usually, our test confirms that about 70% of insects can transmit RGDV to healthy rice seedlings after a latent period of 10 days. At 6 days padp, TUNEL-positive signs could be detected in limited areas of virus-infected intestines in about 70% of viruliferous insects, while few TUNEL-positive cells were found in the nonviruliferous insects (Fig 7A and 7B). However, we could not detect specific TUNEL-positive signs in the virus-infected salivary glands of viruliferous insects at 10 days padp. Thus, the RGDV-induced apoptotic response appears to be restricted to a low level to avoid serious damage to *R*. *dorsalis*, similar to results reported previously for arboviruses in mosquito vectors [14, 25–27].

Electron microscopy showed that the epithelial cells of virus-free intestines had normal histology and ultrastructure, including intact and orderly microvilli, evenly distributed chromatin, and abundant mitochondria with tightly involuted cristae (Fig 7C). In contrast, at 10 days padp, morphological changes in the intestinal epithelial cells of viruliferous *R*. *dorsali* were evident, including cytoplasmic reduction and vacuolization, damaged or decreased number of microvilli, and shrunken or crescent-shaped nuclei (Fig 7D and 7E). Furthermore, the degenerated mitochondria with indiscernible cristae were surrounded by bundles of Pns11 fibrillar structures in virus-infected intestines, compared with the intact mitochondria in nonviruliferous control (Fig 7F and 7G). This abnormal cytopathology of virus-infected intestinal epithelium further suggested that RGDV infection also induces typical mitochondrial-dependent apoptotic response in intact insect vectors.

### Apoptotic response regulates RGDV infection in intact insect vectors

**Fig 8:**
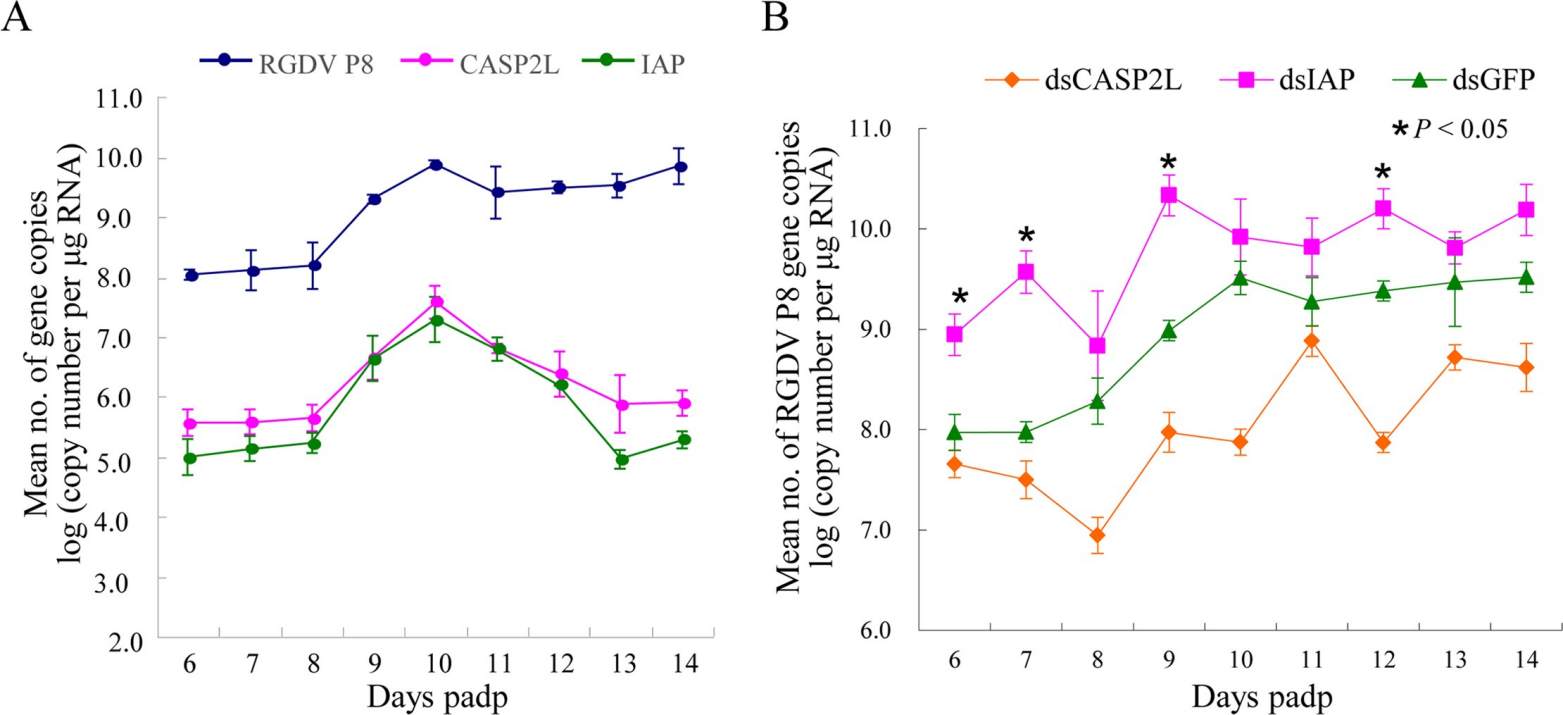
The apoptotic response regulated the persistent RGDV infection of *R*. *dorsalis*. (A) Mean number copies for RGDV P8, CASP2L or IAP genes in viruliferous leafhoppers from 6 to 14 days padp as determined by RT-qPCR assay. Means (±SD) from three independent biological replicates are shown. (B) Mean number of viral genome copies in dsCASP2L-treated, dsIAP-treated or dsGFP-treated viruliferous leafhopper from 6 to 14 days padp. Means (±SD) from three independent biological replicates are shown. Statistical significance is related to the dsGFP control. *P < 0.05.

To further investigate the effects of apoptotic response on viral infection in insect vectors, from 6 to 14 days padp, we sampled 30 live viruliferous leafhoppers daily and then examined the gene expression profiles for apoptosis-related factors (CASP2L and IAP) and major outer capsid protein P8 of RGDV by RT-qPCR assay. During the latent period for RGDV in its insect vectors, before 10 days padp [42], the transcript levels of apoptosis-related genes increased, and then decreased (Fig 8A). Similarly, the transcript level of viral major outer capsid protein P8 gene also increased quickly to peak at 10 days padp, and then remained relatively stable (Fig 8A). Although changes in the relative expression of apoptosis-related genes are not informative regarding the biology of apoptosis in an insect, our results suggest a positive association between viral infection and the expression of apoptosis-related genes in the insect vector.

We then manipulated apoptotic response using RNAi to explore the role of apoptosis during viral infection in insect bodies. Viral titers in 30 live viruliferous leafhoppers after dsRNAs microinjection were examined daily. We tested the efficiency of the knockdown of the targeted genes after 2 days post microinjection of dsRNAs in intact insects (S5B Fig). Time-course experiments showed that the profiles of mean number of RGDV P8 gene copies in all dsRNAs treatments were similar (Fig 8B). From 6 to 14 days pdap, the mean number of RGDV P8 gene copies in the dsIAP, dsCASP2L and dsGFP treatment was 5.45 × 10^9^, 1.01 × 10^8^ and 8.53 × 10^8^ copies/μg insect RNA, respectively (Fig 8B). These results demonstrated that blocking the apoptotic pathway inhibited viral infection, while promoting the apoptotic pathway facilitated viral infection. Thus, we concluded that RGDV induced and utilized apoptosis for viral infection in *R*. *dorsalis* vectors.

We then calculated the mortality of 100 viruliferous or nonviruliferous insects daily after dsRNA microinjection. Our preliminary test indicated that the microinjection itself had little effect on the mortality rates of nonvirulifeorus *R*. *dorsalis* adults (S6A Fig). For both nonviruliferous and viruliferous insects, the mortality of dsIAP-treated *R*. *dorsalis* was higher than those treated with dsGFP or dsCASP2L. For example, at 6 days post microinjection, approximately 43.0% of the dsIAP-treated viruliferous insects were dead, compared with mortality rates of about 33.0% for the dsGFP-treated and about 24.0% for dsCASP2L-treated viruliferous insects (S6B Fig). However, at the same number of days post microinjection, the mortality rates for the dsCASP2L-, dsGFP- and dsIAP-treated nonviruliferous insects were approximately 11.3%, 16.3% and 26.0%, respectively (S6B Fig), supporting the fact that IAP is necessary to maintain cell viability in insects [43]. Furthermore, our attempt to silence IAP resulted in a simultaneous increase in apoptotic response and death in viruliferous *R*. *dorsalis*, illustrating the possible positive association between virus-induced apoptotic response and insect mortality.

## Discussion

Many important plant viruses are persistently transmitted via insect vectors with limited harm to the insects. Here, we used the plant reovirus RGDV and leafhopper vector *R*. *dorsalis* to determine how the apoptotic response was activated during persistent viral transmission by insect vectors. We first demonstrated that RGDV infection induced typical apoptotic characteristics in cultured leafhopper cells, including degeneration of mitochondria, decrease in mitochondrial membrane potential, and appearance of condensed chromatin, chromosomal DNA fragments and virus-containing apoptotic bodies (Figs 1 and 2), verifying that RGDV triggered the typical apoptotic response in insect vector cells. The expression of caspases (CASP2L and CASP8L) and IAP genes were up-regulated during viral infection in cultured leafhopper cells (Fig 5). The knockdown of caspase gene expression using RNAi blocked apoptotic response and led to the significant inhibition of synthesis of viral mRNAs and genome RNAs, and the accumulation of viral proteins in insect vector cells (Figs 5 and 6). However, the knockdown of IAP gene expression using RNAi promoted apoptotic response, causing a significant increase of these viral replication processes in insect vector cells (Figs 5 and 6). Thus, RGDV exploit the apoptotic response in a caspase-dependent pathway to promote viral replication in insect vector cells. In insect bodies, RGDV infection also triggered a similar apoptotic response restricted to a low level, which appeared to benefit viral replication, but may have damaged functionally relevant tissues and organs, decreasing the fitness of the vectors (Figs 7 and 8). Thus, the typical apoptotic response can be induced and facilitated viral accumulation during viral replication and transmission by the insect vectors.

As we observed for the RGDV–*R*. *dorsalis* combination, the similar apoptotic response is also restricted to a low level in mosquito vectors [27]. In general, the induction of apoptosis promoted viral infection but also harmed the insects. Thus, viruses have evolved some mechanisms to avoid stimulating extensive apoptotic responses in the bodies of insect vectors. During the latent period for RGDV in insect vectors, before 10 days padp [42], the expression levels of apoptosis-related genes (CASP2L, CASP8L and IAP) increased, then decreased (Fig 8A), indicating that the apoptotic response was activated during replication and then was suppressed. Such synchronous gene expression for CASP2L, CASP8 and IAP suggested that virus-induced apoptotic response was critically modulated during viral infection of insect vectors. For controlling the excessive viral accumulation and avoiding obvious pathology, IAP, the inhibitor of apoptosis was induced by viruses to restrict the apoptotic response to a low level. Other as-yet unknown anti-apoptotic mechanisms might also be activated to block or restrict apoptotic response. Furthermore, a conserved siRNA antiviral response was triggered by RGDV infection to control viral propagation, avoiding excessive viral accumulation past the pathogenic threshold in insect vectors [24]. Previously, we also found that cellular structures and pathways such as microtubules, intermediate filaments or autophagy were induced by RGDV infection, promoting viral infection but also causing some insect cytopathology [44, 45]. It appeared that all these mechanisms were involved in modulating a metastable balance between viral accumulation and adverse effects, allowing the virus to be persistently transmitted by insect vectors.

The multifunctional mitochondrion not only plays an essential role in host immune responses but also serves as an important control point in the regulation of apoptosis [7]. After apoptotic signaling, the mitochondrial membrane potential is lost, and apoptosis-related factors are released [8–10]. Numerous viral proteins, including Vpr protein of human immunodeficiency virus (HIV), X protein of hepatitis B virus (HBV), PB1-F2 of influenza A virus (IAV), and NS4A of hepatitis C virus (HCV), have been reported to directly target mitochondria to activate mitochondrial apoptosis in mammalian host cells [46–50]. VDAC, an outer membrane protein of mitochondria, is often activated during apoptosis and is targeted by viral proteins to initiate apoptosis [46, 51–52]. In fact, VDAC is a porin channel that serves as a major diffusion pathway for ions and metabolites to control mitochondrial membrane permeabilization [39]. Our data revealed that the fibrillar structures composed of nonstructural protein Pns11 of RGDV could target the mitochondria, induce mitochondrial degeneration and decrease the mitochondrial membrane potential in the absence of viral replication (Figs 3 and 4), suggesting that RGDV Pns11 was responsible for initiating virus-induced apoptotic response. Such attachment of virus-induced fibrillar structures with mitochondria possibly was mediated by the specific interaction of RGDV Pns11 with the VDAC (Figs 3 and 4). How the association of VDAC with Pns11 of RGDV is involved in the induction of apoptotic response during viral replication in insect vectors is still unknown. Our study is the first to directly confirm that a nonstructural protein encoded by a persistent-propagative plant virus induce the apoptotic response in an insect vector to promote viral infection and transmission.

Based on the results described, we propose that RGDV exploits the apoptotic mechanism for efficient infection in insect vector cells. However, there are still many unknowns in the apoptotic process. For example, how does RGDV trigger and exploit the apoptotic response for efficient infection and how does it evolve such a strategy to enable persistent infection? In some cases, viruses may directly exploit apoptotic bodies for their dissemination and subsequent infection of a mammalian host or an insect vector [9, 12, 53, 54]. In our study, whether packaging of the RGDV virions within apoptotic bodies protects them from insect immune mechanisms was not determined. In insect vectors, one possible consequence of apoptosis is that physical barriers within the insect are weakened. We thus deduced that the apoptotic response is exploited by RGDV to overcome multiple tissue and membrane barriers to enable efficient infection of its leafhopper vectors. In addition, we still do not know how virus-induced apoptotic response is restricted to a low level in insect vectors. New approaches based on the reverse genetics systems for plant reoviruses and on CRISPR/Cas9 technologies for the leafhopper vector, combined with continuous insect vector cell lines will provide new opportunities to unravel the molecular mechanisms for virus-induced apoptotic response in the RGDV–*R*. *dorsalis* system.

## Materials and methods

### Insects, cells, viruses and antibodies

Nonviruliferous individuals of the leafhopper *R*. *dorsalis* were collected from Guangdong Province in southern China. The continuous cultured cell line derived from *R*. *dorsalis* was originally established from embryonic fragments dissected from insect eggs and maintained on growth medium as described previously [17]. The *R*. *dorsalis* cell line supported a uniform and synchronous viral infection, enabling the early viral replication process to be traced [42]. RGDV was purified from infected cultured insect vector cells as described previously, and resuspended in His-Mg buffer (0.1 M histidine, 0.01 M MgCl_2_, pH 6.2) [17]. Synchronous infection of continuous cultured cells by RGDV was initiated as described by Wei et al. [55]. When the cultured monolayer of leafhopper cells on a coverslip (15 mm diameter) reached 80% confluency, cells were inoculated with purified RGDV at a MOI of 0.1 or 1 for 2 h, washed twice, and covered with growth medium at 25°C. His-Mg buffer-treated cells served as controls. Rice samples infected with RGDV were initially collected from Guangdong Province. Rabbit polyclonal antisera against intact viral particles, major outer capsid protein P8, and nonstructural proteins Pns11 and Pns12 were prepared as described previously [29, 32, 36]. IgGs were purified from specific polyclonal antisera, then conjugated to rhodamine or fluorescein isothiocyanate (FITC), according to the manufacturer’s instructions (Thermo Fisher). The antibody against cytochrome c was obtained from BD Biosciences.

### Recombinant baculovirus expressing RGDV Pns11

Sf9 cells infected with recombinant baculovirus vector containing Pns11 of RGDV have been previously described [17]. In brief, the coding region of the ORF for RGDV Pns11 was amplified by PCR. The purified product was cloned into Gateway vector pDEST8 (Thermo Fisher) to construct plasmid pDEST8-Pns11. Then the recombinant baculovirus vector was introduced into *E*. *coli* DH10Bac (Thermo Fisher) to generate a recombinant bacmid. The isolated recombinant bacmid was used to transfect Sf9 cells in the presence of Cellfectin II (Thermo Fisher) according to the manufacturer’s instructions. After a high-titer baculoviral stock was generated, amplification of the viral stock was scaled up to an appropriate volume for cellular infection on coverslips or in flasks. At 48 hpi, Sf9 cells, growing on coverslips and infected with recombinant bacmids, were treated for immunoelectron microscopy. Sf9 cells inoculated with empty baculovirus vector served as negative controls. They were also fixed, permeabilized, and immunolabeled with Pns11-specific IgGs conjugated to FITC (Pns11-FITC) for immunofluorescence microscopy of Pns11 overexpression and mitochondria.

Sf9 cells, growing in flasks and infected with recombinant bacmids, were harvested for cell viability tests using 0.4% trypan blue solution at 72 hpi. Cell images and counts were made in an automated cell counter (Counter Star).

### Electron microscopy

Virus-infected cultured *R*. *dorsalis* cells growing in a monolayer on coverslips and intestines dissected from viruliferous *R*. *dorsalis* or Sf9 cells were fixed, dehydrated, and embedded, and ultrathin sections were cut as previously described [31]. For immunoelectron microscopy, sections were immunolabeled with the Pns11-specific IgGs as the primary antibody, followed by treatment with goat anti-rabbit IgG conjugated with 15-nm-diameter gold particles as the secondary antibody (Abcam), as previously described [31].

### Measurement of mitochondrial membrane potential

The MitoProbe JC-1 Assay Kit for Flow Cytometry (Thermo Fisher) was used to measure the change in mitochondrial membrane potential in cultured cells of *R*. *dorsalis* at 48 hpi. Briefly, approximately 1 × 10^6^ cells were collected and suspended in 1 mL PBS. JC-1 was added to cells at a final concentration of 2 μM, and after incubation at 37°C for 30 min, cells were washed once with warm PBS, then resuspended in PBS and immediately examined with a flow cytometer (BD FACS Calibur). Data from three independent biological experiments were analyzed using Cellquest software and displayed as a dot plot of JC-1 green fluorescence (*x*-axis) against red fluorescence (*y*-axis).

Changes in mitochondrial membrane potential in Sf9 cells infected with the recombinant baculovirus expressing Pns11 were measured using rhodamine 123 fluorescence (Thermo Fisher) at 72 hpi. Sf9 cells inoculated with empty baculovirus vector served as a negative control. In brief, approximately 1 × 10^6^ Sf9 cells were harvested and suspended in PBS, then incubated with rhodamine 123 at a concentration of 1 μM in PBS at 37°C for 1 h. Cells were washed with PBS, then resuspended in PBS and analyzed immediately using the flow cytometer. Data from three independent biological experiments were analyzed and displayed as a plot of fluorescence intensity of rhodamine (*x*-axis) against cell number (*y*-axis).

### DNA fragmentation assay

At 72 hpi, cultured cells of *R*. *dorsalis* were harvested, and DNA was extracted using the Cell Apoptosis DNA Ladder Detection Kit (KeyGEN BioTECH), according to the manufacturer’s instructions. Chromosomal DNA fragments were separated using 2% agarose gel electrophoresis, and DNA ladders were visualized by ethidium bromide staining.

### TUNEL assay

*R*. *dorsalis* cells cultured in a monolayer on coverslips were fixed in 4% v/v paraformaldehyde and treated with 0.2% v/v Triton-X, as previously described [56]. Then the DeadEnd Fluorometric TUNEL System (Promega) was used for TUNEL staining. According to the manufacturer’s instructions, samples were treated with the equilibration buffer in the kit at room temperature for 10 min, then incubated with rTdT incubation buffer at 37°C for 60 min. Thereafter, the reaction was terminated by adding 2× sodium citrate in the kit, and then incubated with viral particle-specific IgG conjugated to rhodamine (virus-rhodamine). Nick-end-labeling of nucleosome fragments with fluorescein-dUTP and viral infection were visualized using a confocal microscope.

For observing TUNEL signals during viral infection of insect vectors, second instars of *R*. *dorsalis* were fed on diseased rice plants for 2 days and then transferred to healthy rice seedlings. At different days padp, the intestines or salivary glands were dissected and processed for TUNEL assay, as described above. Meanwhile, samples were immunolabeled with virus-specific IgGs conjugated to rhodamine (virus-rhodamine) and actin dye phalloidin-Alexa Fluor 647 carboxylic acid (Invitrogen), then processed for immunofluorescence microscopy as described previously [17]. Three independent biological replicates were conducted and analyzed.

### Immunofluorescence microscopy of mitochondria

Virus-infected cultured *R*. *dorsalis* cells or Pns11-expressing Sf9 cells growing on coverslips were incubated with MitoTracker Red CMXRos (Thermo Fisher) for 45 min using standard growth conditions. After the staining solution was carefully removed, cells were fixed in 4% v/v paraformaldehyde and permeabilized in 0.2% v/v Triton-X, immunolabeled with Pns11-FITC, then examined with immunofluorescence microscopy.

To set up the immunofluorescence microscopy parameters, digital images (1024×1024 pixels) were captured with either 488 nm excitation (emission filters) or 543 nm excitation. They were acquired with a 63 oil-immersion objective. Samples in the same group possessed the same parameters of immunofluorescence microscopy to unitize the background.

### Y2H assay

A Matchmaker Gold Yeast-two-hybrid system (Clontech, USA) was used for Y2H screening. The cDNA library derived from *R*. *dorsalis* or the VDAC gene was constructed in the pGADT7 vector for prey plasmids. Full-length ORF of RGDV Pns11 was cloned in the pGBKT7 vector as a bait plasmid, which was then used to transform yeast strain AH109 to confirm the absence of self-activation and toxicity. Thereafter, the prey and bait were used to cotransform AH109, and transformants were screened on the SD double-dropout (DDO) medium (SD/-Leu/-Trp), SD triple-dropout (TDO) medium (SD/-His/-Leu/-Trp) and SD QDO medium (SD/-Ade/-His/-Leu/-Trp). Positive clones were selected on QDO/X plates containing X-α-Gal (20 μg/mL) to detect β-galactosidase activity. The interaction of pGBKT7-53 with pGADT7-T served as a positive control and that of pGBKT7-Lam with pGADT7-T served as a negative control.

We then used the DUALmembrane starter kit (Dualsystems Biotech) to detect the interaction between membrane-associated VDAC and RGDV Pns11 according to the manufacturer’s instructions. Full-length ORF of RGDV Pns11 or VDAC was inserted into bait vector pBT3-STE or prey vector pPR3-N. Thereafter the bait and prey were used to transform the yeast strain NMY51, and transformants were screened on the TDO and QDO medium. The clones were streaked on QDO/X plates containing X-Gal for color formation in a β-galactosidase assay. The pTSU2-APP/ pNubG-Fe65 interaction served as a positive control, and the pTSU2-APP/ pRR3N served as a negative control.

### GST pull-down assay

A GST pull-down assay was performed as previously described [57]. The Pns11 gene of RGDV was cloned in the pGEX-3x vector to construct a plasmid expressing the GST fusion protein as a bait (GST-Pns11). The full-length ORF of the VDAC from *R*. *dorsalis* was cloned into the pHM4 vector to construct a plasmid expressing the His fusion protein as a prey (His-VDAC). Recombinant proteins GST-Pns11 and GST were respectively expressed in the *Escherichia coli* stain BL21. Lysates were then incubated with glutathione-Sepharose beads (Amersham) and subsequently, with the recombinant protein His-VDAC. Finally, eluates were analyzed using GST-tag and His-tag antibodies (Sigma), respectively, in a Western blot assay.

### Apoptotic response activation in response to viral infection

Cultured *R*. *dorsalis* cells in a monolayer with 80% confluency were inoculated with RGDV at a MOI of 1.0 for 2 h. His-Mg buffer-treated cells served as controls. At 48 hpi, the cells were collected at the same time. For viral acquisition by insects, about 500 nonviruliferous second instar nymphs of *R*. *dorsalis* were fed on RGDV-infected rice plants for 2 days, then transferred to healthy rice seedling. From 6 to 14 days padp, 30 leafhoppers were daily collected at the same time.

Total RNA was extracted from cells or insects using TRIzol Reagent (Thermo) according to the manufacturer’s instructions. For synthesizing first-strand cDNA, total RNA was primed with oligo-dT primer and reverse transcribed with M-MLV Reverse Transcriptase (Promega). The qPCR assays were performed in a Mastercycler Realplex4 real-time PCR system (Eppendorf) using GoTaq qPCR Master Mix kit (Promega) with efficient and specific primers (S1 Table). For relative quantitation, the transcriptional level of the actin gene from the leafhopper was used as the control for each qPCR assay. Relative levels of genes were qualitatively analyzed using the 2^−ΔΔCt^ method. For absolute quantification, the number of RGDV P8 gene copies and CASP2L and IAP gene copies were calculated as the log of the copy number/μg insect RNA based on a standard curve of the RGDV P8 gene, CASP2L gene and IAP gene, respectively.

### Effect of synthesized dsRNAs or Z-VAD-FMK on viral infection in cultured cells of *R*. *dorsalis*

The T7 RNA polymerase promoter was added to the forward primer and reverse primer at the 5′ and 3′ terminal to amplify a region of about 500–900 bp in each gene (S1 Table). PCR products were transcribed into dsRNAs *in vitro* using the T7 RiboMAX (TM) Express RNAi System, according to the manufacturer’s protocol (Promega). Purified dsRNAs were examined using agarose gel electrophoresis to determine their integrity and quantified by spectroscopy. Three microliters of Cellfectin II Reagent (Thermo) and 4 μg dsGFP, dsVDAC, dsCASP2L, dsCASP8L or dsIAP were diluted individually in 25 μL LBM without antibiotics and fetal bovine serum, and mixed gently together at room temperature for 20 min. Then the dsRNA–lipid complex was incubated with the cultured cells of *R*. *dorsalis* in a monolayer (at 80% confluency) for 8 h. Thereafter, cells were inoculated with purified RGDV (MOI of 0.1) for 2 h. Infected cells were processed for immunofluorescence or were harvested for RT-qPCR detection at 48 or 72 hpi. Alternatively, at 72 hpi, total proteins were extracted from infected cells and further analyzed by immunoblotting with antibodies against P8 and Pns12, respectively. Insect actin was detected with actin-specific antibodies (Sigma) as a control.

Furthermore, viral genome dsRNAs were isolated from cultured cells, as described previously [58]. In brief, viral genome dsRNAs from cell lysates were isolated at 72 hpi using the modified CF11 cellulose chromatography procedure. Cell lysates of each dsRNA treatment were mixed with 1× STE (0.1 M NaCl, 0.05 M Tris, 0.001 M EDTA, pH 6.8), 10% SDS, and 2× STE-saturated phenol. Following centrifugation, the aqueous phase was recovered and added the ethanol to a final volume of 16.5%. Then CF11 cellulose (Whatman) was loaded and vortexed to mix thoroughly. The pellets after the centrifugation were washed with 1×STE in 16.5% ethanol. Finally, the dsRNAs were eluted from CF11 with 1×STE. Separation of genomic dsRNA segments were loaded on 1.0% agarose gel. The pattern of genomic dsRNA segments separated from purified RGDV virons was considered as the reference [41].

For the northern blots, at 72 hpi, the total RNAs from cultured cells were extracted with TRIzol Reagent (Thermo Fisher). The DIG High Prime DNA labelling and Detection Starter KitⅠ(Roche) was used to examine the transcript level of RGDV P8 and Pns12. In brief, about 500 bp DIG-labeled DNA probe of P8 or Pns12 about were generated after the incubation of denatured PCR products (S1 Table) and DIG-High Prime for 20 h at 37°C. About 5 μg total RNA of each dsRNA treatment were loaded and detected for the transcript level of P8 or Pns12. The 5.8S rRNA stained with Methylene blue were served as a control to confirm loading of equal amounts of RNA in each lane.

In addition, the relative abundance of CASP2L, CASP8L or IAP genes in virus-free cultured cells of *R*. *dorsalis* after different times of transfection with dsRNAs was quantified by RT-qPCR as described already. Three independent biological replicates were conducted and analyzed.

Cultured *R*. *dorsalis* cells were treated for 4 h with 25 μM pancaspase inhibitor Z-VAD-FMK (Promega) dissolved in DMSO. Cells were treated with DMSO as the control. Cells were then inoculated with purified virus (MOI of 0.1), then assayed by RT-qPCR at 48 hpi, as described above. Three independent biological replicates were conducted and analyzed.

### Effect of synthesized dsRNAs on viral infection and insect mortality

Generally, first or second instar nymphs of *R*. *dorsalis* are the most efficient stage for acquiring RGDV from infected rice plants [19]. Furthermore, RGDV takes at least 2 days to infect the intestinal epithelium of *R*. *dorsalis*, so the synthesized dsRNAs are microinjected directly into the insect abdomen for efficient dsRNA delivery instead of oral feeding [59]. We first allowed 700 nonviruliferous second instar nymphs to feed on RGDV-infected rice plants for 2 days to acquire viruses, then microinjected them with synthesized dsGFP, dsCASP2L or dsIAP (about 0.05 μg/insect) using a Nanoject II Auto-Nanoliter Injector (Spring). The treated insects were transferred to healthy rice seedlings until they were assayed. Viral titers in 30 live leafhoppers treated with dsRNAs were assayed daily by RT-qPCR for viral gene copies of the major outer capsid protein P8. The equation of y = -3.349x +49.258 (y = the logarithm of plasmid copy number to base 10, x = Ct value, R^2^ = 0.9995) was used to calculate the viral genome copy as the log of the copy number per microgram of insect RNA [60]. For calculating mortality of insects, 100 insects of each dsRNA treatment were individually fed on a healthy rice seedling in one glass tube after microinjection. The dead insects were counted at the same time each day. The mortality rate was calculated as the number of dead dsRNAs-treated insects in the total number of dsRNAs-treated insects. In addition, the relative abundance of CASP2L and IAP genes in 10 nonviruliferous leafhoppers was also estimated by RT-qPCR assay. Three independent biological replicates were conducted and analyzed.

### Statistical analyses

All data for cultured cells and some data from insects, including percentage of TUNEL-positive intestines and relative expression of genes, were analyzed with a two-tailed *t*-test in GraphPad Prism 7. The data for insect mortality were analyzed using SPSS, version 17.0. Percentage data were arcsine square-root-transformed before analysis. Multiple comparisons of the means were performed using Tukey’s honestly significant difference (HSD) test and a one-way analysis of variance (ANOVA). Data were back-transformed after analysis for presentation in the text and figures.

## Supporting information

S1 Fig RGDV infection caused a slight cytopathological change in continuous cultured cells of *R. dorsalis*. Cells were mock-infected or infected with purified RGDV virions (MOI of 1). His-Mg buffer-treated cells served as mock controls. Cells were photographed at 72 hpi. Bars, 20 μm.
(TIF)

S2 Fig RGDV Pns11 alone reduced viability of Sf9 cells and caused release of cytochrome c from mitochondrial. (A) Over-expression of RGDV Pns11 reduced cell viability of Sf9. At 72 hpi, Sf9 cells growing in a flask were suspended and stained with trypan blue solution, then analyzed with the cell counter. Cell concentration of Pns11-expressing cells was lower than that of the mock. Bars, 40 μm. (B) Viability of Pns11-expressing cells was lower than that of the mock. Means (±SD) from three biological replicates are shown. **P < 0.005. Data were analyzed with a two-tailed *t*-test in GraphPad Prism 7. (C) Translocation of cytochrome c to cytosol occurred in Pns11-expressing cells at 72 hpi as determined by immunofluorescence assay. Mock- or Pns11-expressing Sf9 cells were immunostained with cytochrome c (green) and MitoTracker (red). Bars, 10 μm. Sf9 cells inoculated with empty baculovirus vector served as mock controls.
(TIF)

S3 Fig Characterization of VDAC protein sequences. (A) Phylogenetic relationships of VDAC orthologs of *R*. *dorsalis* with counterparts. The available sequences were aligned using Clustal W, and phylogenetic trees were reconstructed by neighbor-joining analysis with *P*-distance using MEGA 5.1. Reliability of the phylogenetic trees was estimated by calculating bootstrap confidence limits based on 1000 replicates. (B) Schematic representation of VDAC protein with the domain of the porin3 superfamily and other binding sites.
(TIF)

S4 Fig Phylogenetic relationships of caspase orthologs of *R. dorsalis* with counterparts. The available sequences were aligned using Clustal W, and phylogenetic trees were reconstructed by neighbor-joining analysis with *P*-distance using MEGA 5.1. Reliability of the phylogenetic trees was estimated by calculating bootstrap confidence limits based on 1000 replicates.
(TIF)

S5 Fig RT-qPCR assay showing the knockdown efficiency of synthesized dsRNAs targeting CASP2L, CASP8L, and IAP genes in intact insects and continuous cultured cells of *R. dorsalis*. (A) Relative expression levels of CASP2L, CASP8L and IAP genes in virus-free cultured cells of *R*. *dorsalis* after 24, 48 or 72 h post transfection with dsRNAs. (B) Relative expression of CASP2L and IAP genes in nonviruliferous *R*. *dorsalis* 2, 4, 6 or 8 d post microinjection with dsRNAs. Means (±SD) from three independent biological replicates are shown. *P < 0.005, **P < 0.0005, ***P < 0.0001. Data were analyzed with a two-tailed *t*-test in GraphPad Prism 7.
(TIF)

S6 Fig Mortality profile of dsRNA-treated *R. dorsalis* from 1 to 8 days post microinjection. (A) Mortality profiles of dsGFP-treated nonviruliferous and normal *R*. *dorsalis* adults from 1 to 8 days post microinjection. (B) Mortality profile of dsCASP2L-treated, dsIAP-treated and dsGFP-treated viruliferous or nonviruliferous *R*. *dorsalis* adults from 1 to 8 days post microinjection. Means (±SD) from three independent biological replicates are shown. Statistical significance is related to the dsGFP control of viruliferous insects. *P < 0.05. Data were analyzed using Tukey’s honestly significant difference (HSD) test using SAS version IV (SAS Institute, Cary, NC, USA).
(TIF)

S1 Table Primers used in this study. (DOCX)
