# Supplementary figures and images for: Fibrillar structures induced by a plant reovirus target mitochondria to activate typical apoptotic response and promote viral infection in insect vectors
Source: PLoS Pathog. 2019 Jan 17;15(1):e1007510. doi: 10.1371/journal.ppat.1007510 (PMC6353215; doi:10.1371/journal.ppat.1007510)

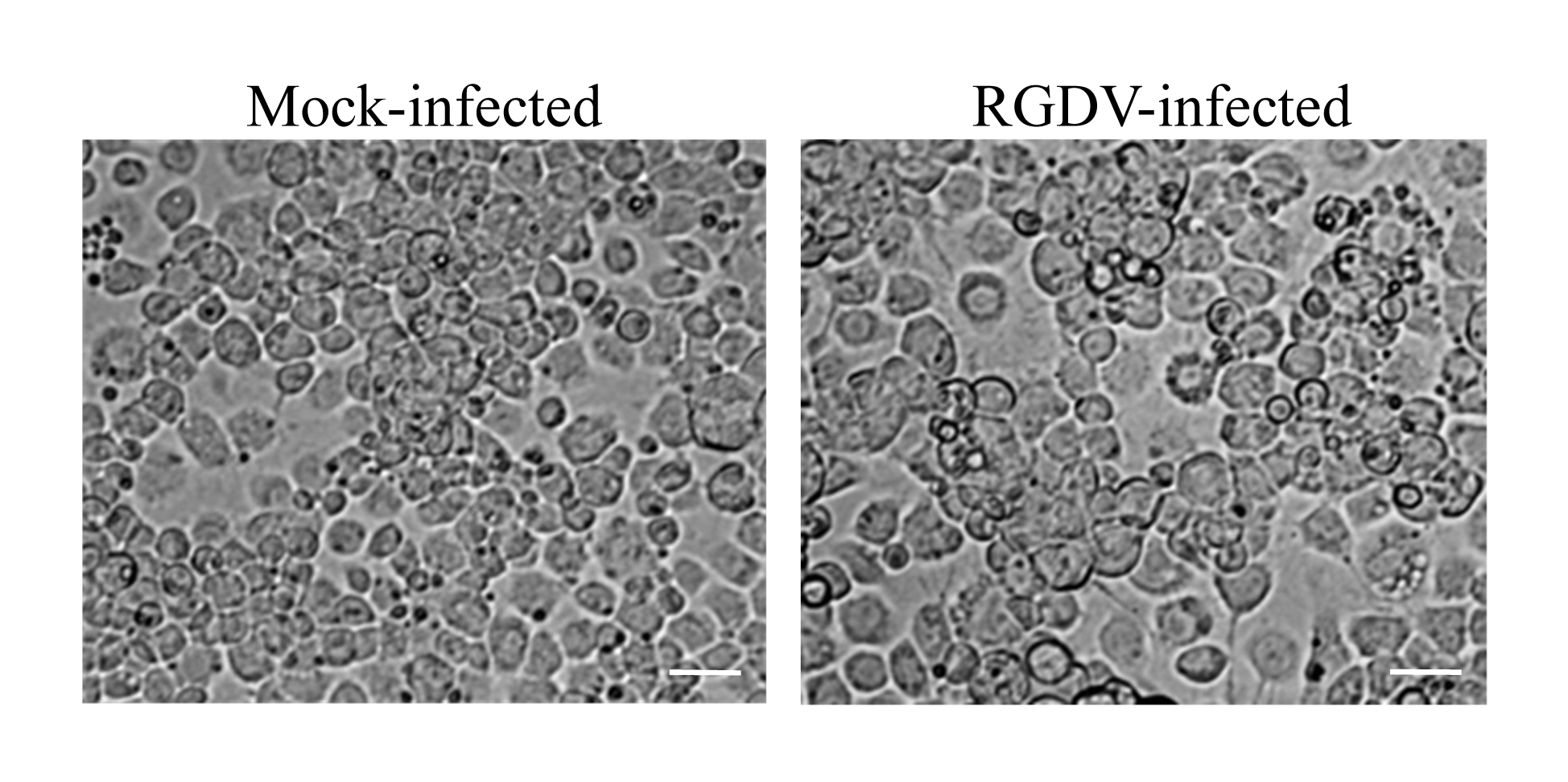

Supplement: S1 Fig — Cells were mock-infected or infected with purified RGDV virions (MOI of 1). His-Mg buffer-treated cells served as mock controls. Cells were photographed at 72 hpi. Bars, 20 μm. (TIF) [file ppat.1007510.s001.tif]

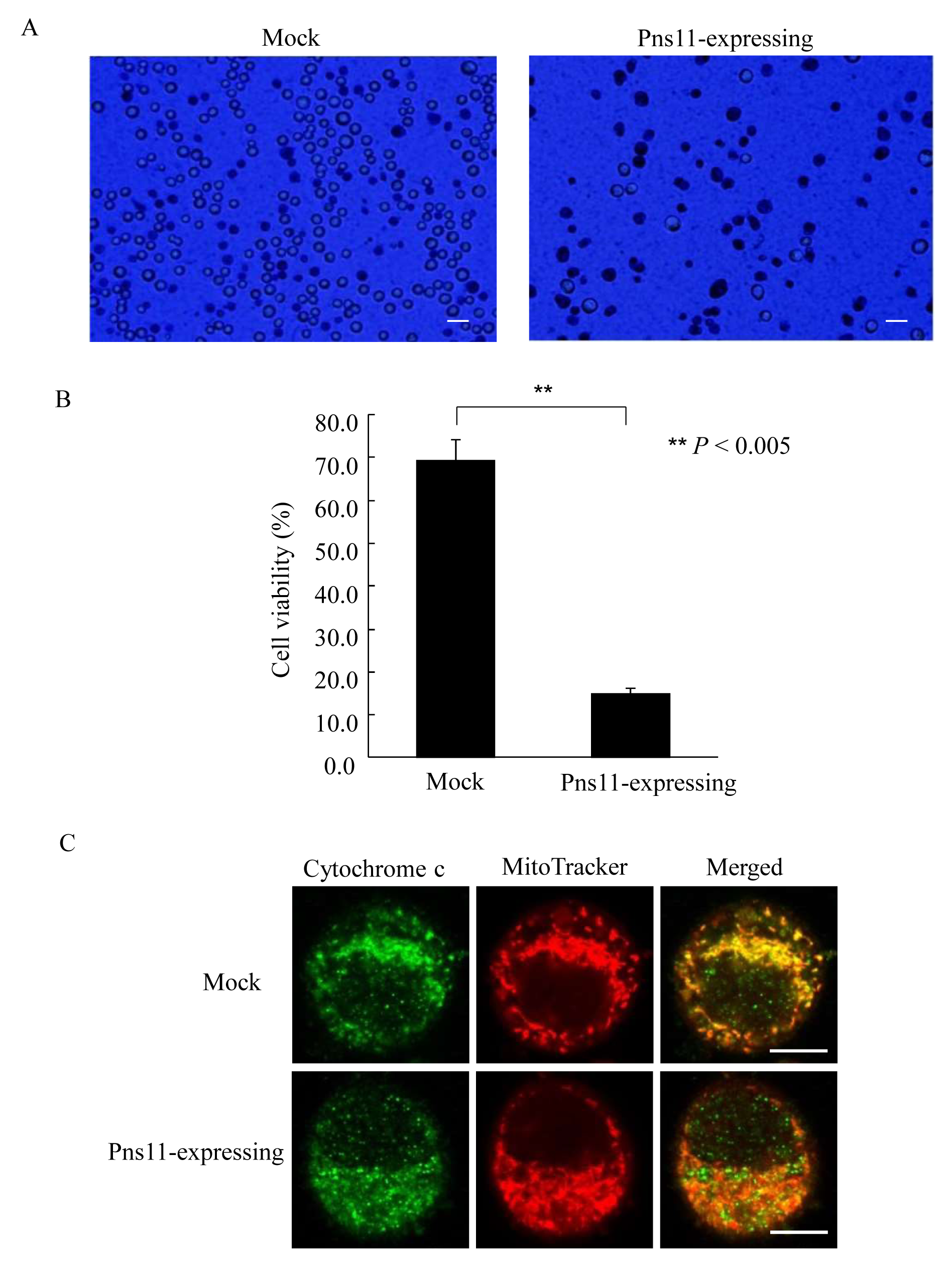

Supplement: S2 Fig — (A) Over-expression of RGDV Pns11 reduced cell viability of Sf9. At 72 hpi, Sf9 cells growing in a flask were suspended and stained with trypan blue solution, then analyzed with the cell counter. Cell concentration of Pns11-expressing cells was lower than that of the mock. Bars, 40 μm. (B) Viability of Pns11-expressing cells was lower than that of the mock. Means (±SD) from three biological replicates are shown. **P < 0.005. Data were analyzed with a two-tailed t-test in GraphPad Prism 7. (C) Translocation of cytochrome c to cytosol occurred in Pns11-expressing cells at 72 hpi as determined by immunofluorescence assay. Mock- or Pns11-expressing Sf9 cells were immunostained with cytochrome c (green) and MitoTracker (red). Bars, 10 μm. Sf9 cells inoculated with empty baculovirus vector served as mock controls. (TIF) [file ppat.1007510.s002.tif]

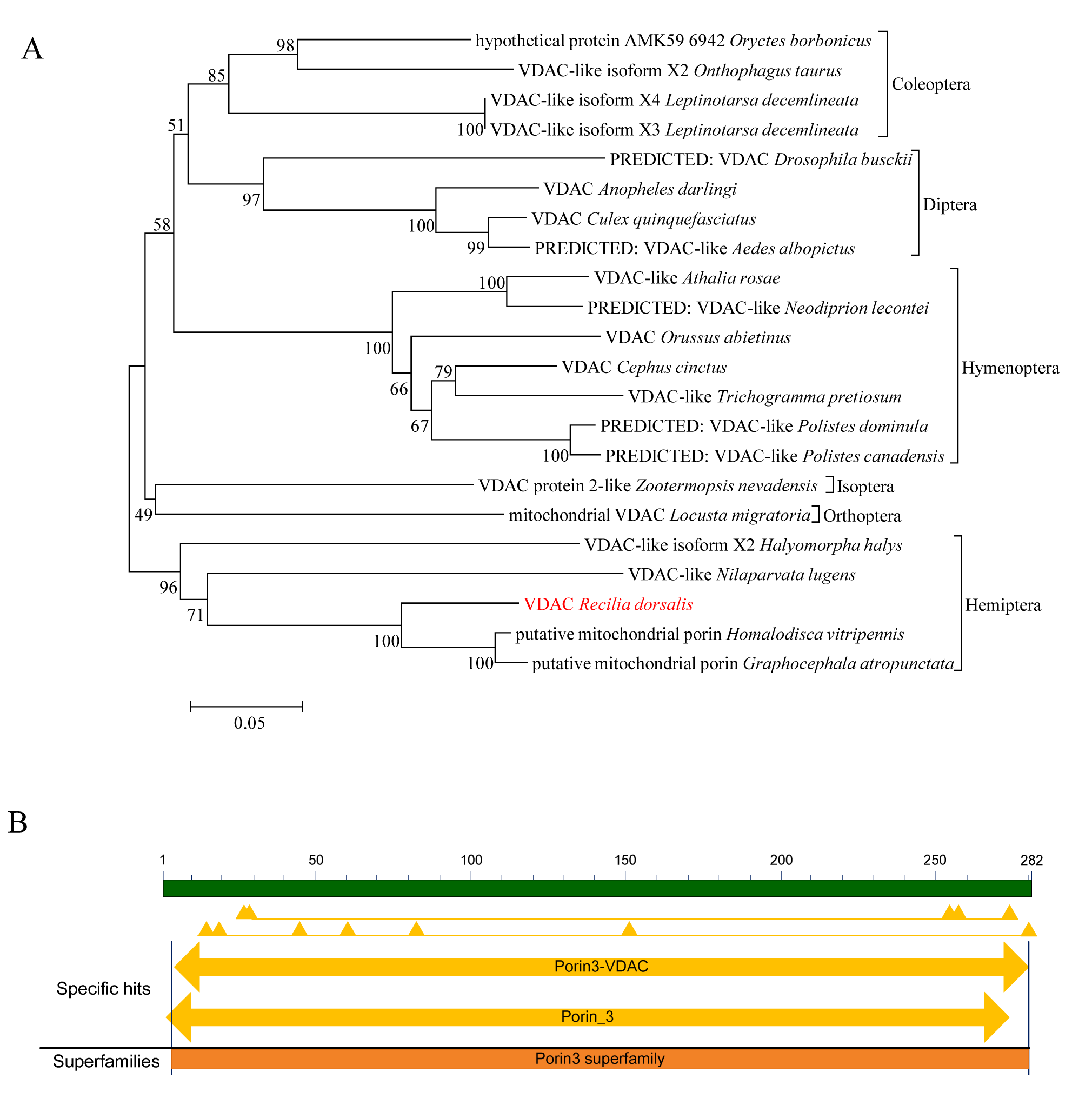

Supplement: S3 Fig — (A) Phylogenetic relationships of VDAC orthologs of R. dorsalis with counterparts. The available sequences were aligned using Clustal W, and phylogenetic trees were reconstructed by neighbor-joining analysis with P-distance using MEGA 5.1. Reliability of the phylogenetic trees was estimated by calculating bootstrap confidence limits based on 1000 replicates. (B) Schematic representation of VDAC protein with the domain of the porin3 superfamily and other binding sites. (TIF) [file ppat.1007510.s003.tif]

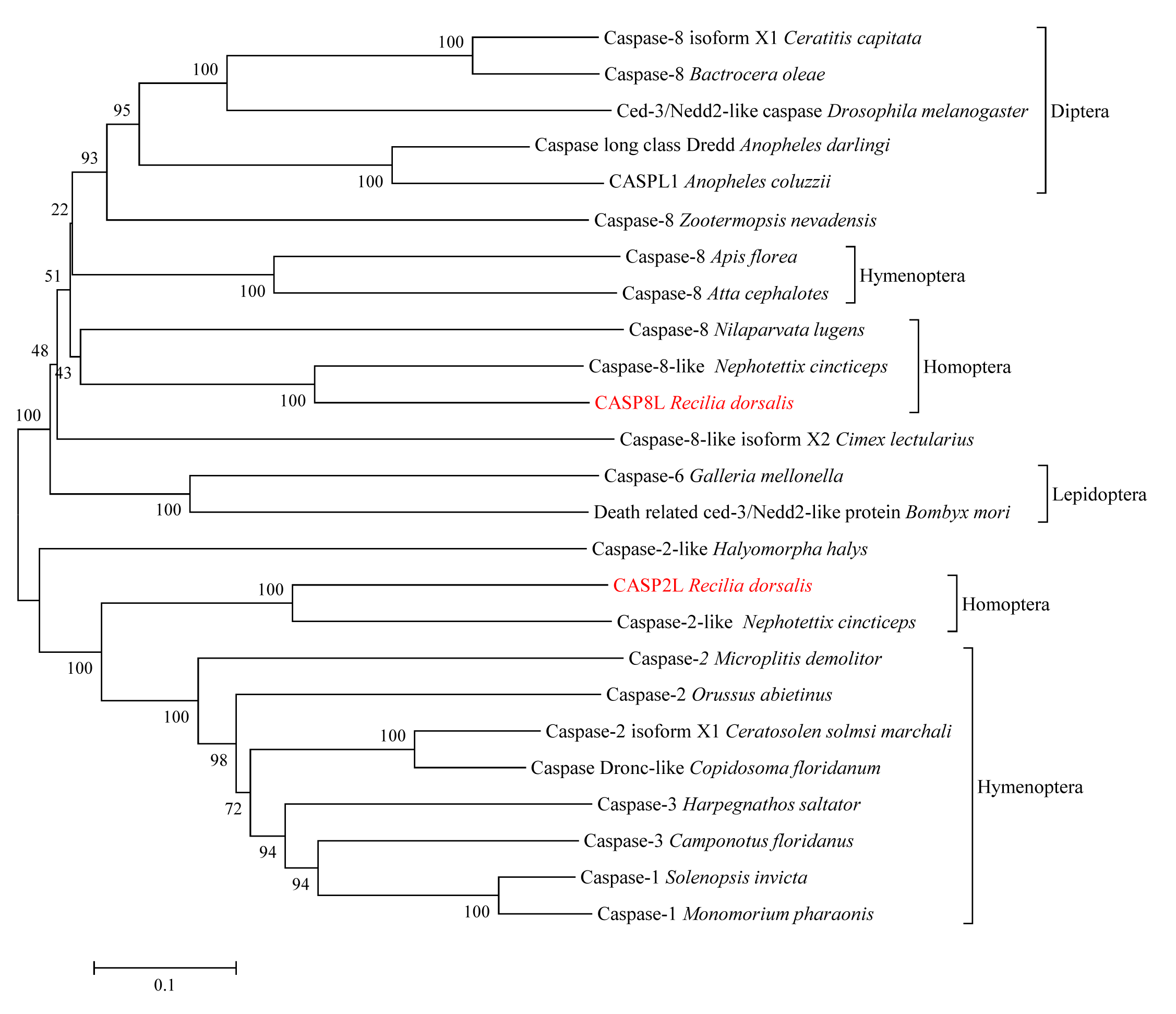

Supplement: S4 Fig — The available sequences were aligned using Clustal W, and phylogenetic trees were reconstructed by neighbor-joining analysis with P-distance using MEGA 5.1. Reliability of the phylogenetic trees was estimated by calculating bootstrap confidence limits based on 1000 replicates. (TIF) [file ppat.1007510.s004.tif]

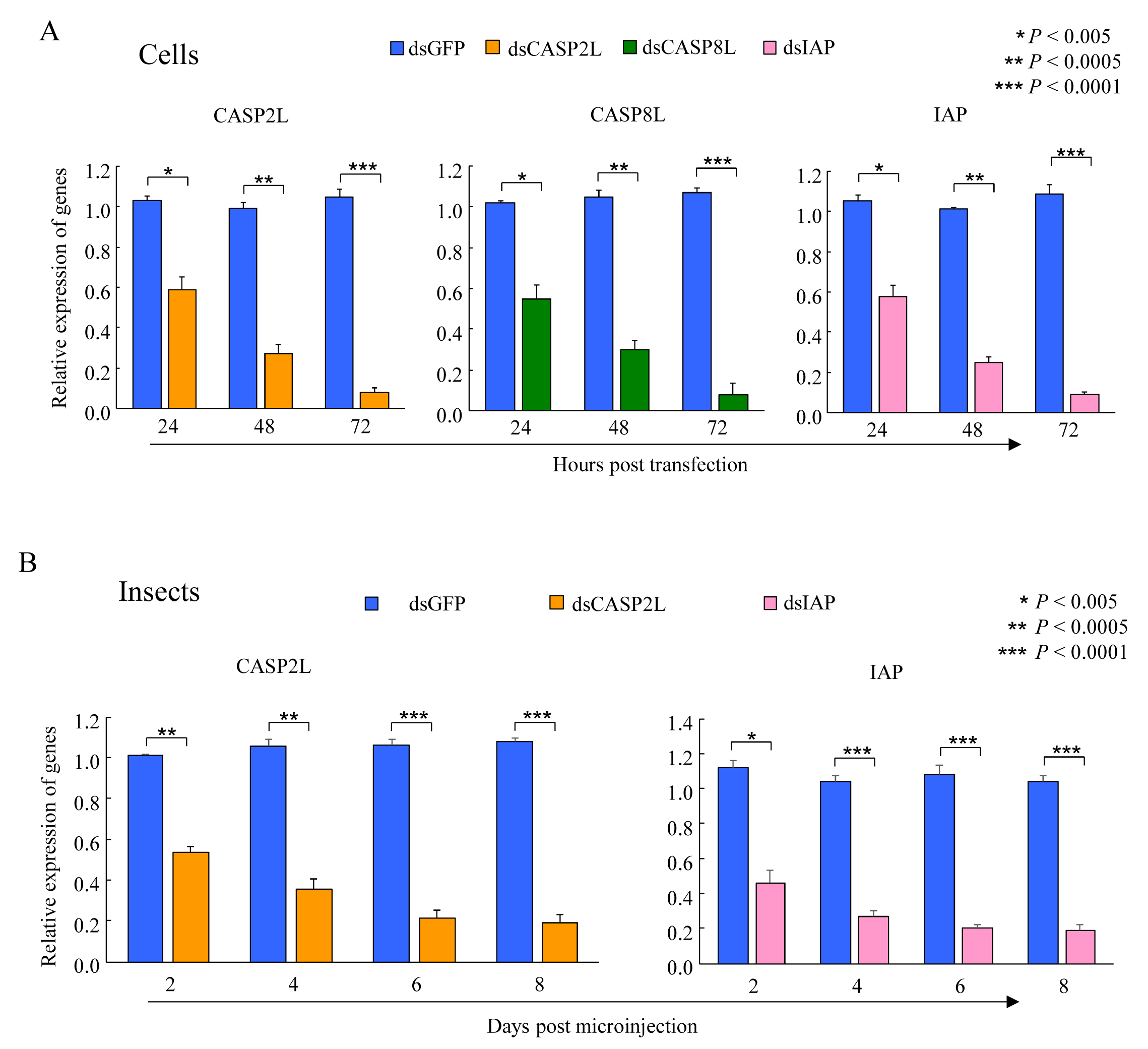

Supplement: S5 Fig — (A) Relative expression levels of CASP2L, CASP8L and IAP genes in virus-free cultured cells of R. dorsalis after 24, 48 or 72 h post transfection with dsRNAs. (B) Relative expression of CASP2L and IAP genes in nonviruliferous R. dorsalis 2, 4, 6 or 8 d post microinjection with dsRNAs. Means (±SD) from three independent biological replicates are shown. *P < 0.005, **P < 0.0005, ***P < 0.0001. Data were analyzed with a two-tailed t-test in GraphPad Prism 7. (TIF) [file ppat.1007510.s005.tif]

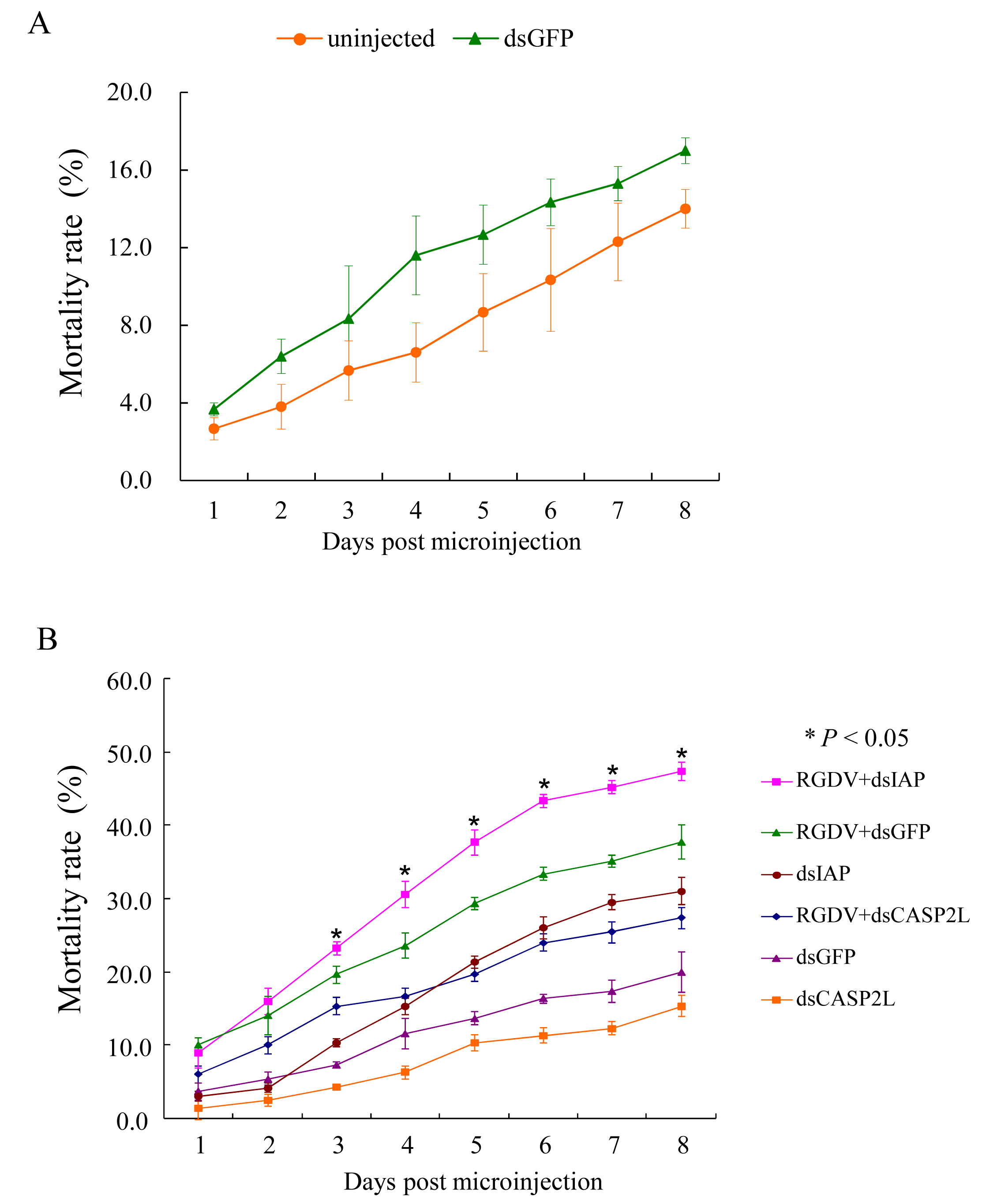

Supplement: S6 Fig — (A) Mortality profiles of dsGFP-treated nonviruliferous and normal R. dorsalis adults from 1 to 8 days post microinjection. (B) Mortality profile of dsCASP2L-treated, dsIAP-treated and dsGFP-treated viruliferous or nonviruliferous R. dorsalis adults from 1 to 8 days post microinjection. Means (±SD) from three independent biological replicates are shown. Statistical significance is related to the dsGFP control of viruliferous insects. *P < 0.05. Data were analyzed using Tukey’s honestly significant difference (HSD) test using SAS version IV (SAS Institute, Cary, NC, USA). (TIF) [file ppat.1007510.s006.tif]
