# Supplementary material for: Fibrillar structures induced by a plant reovirus target mitochondria to activate typical apoptotic response and promote viral infection in insect vectors
Source: PLoS Pathog. 2019 Jan 17;15(1):e1007510. doi: 10.1371/journal.ppat.1007510 (PMC6353215; doi:10.1371/journal.ppat.1007510)
Supplement: S1 Table — (DOCX) [file ppat.1007510.s007.docx]

**S1 Table. Primers used in this study**

| Gene | Primer | Sequence (5′-3′) |
| --- | --- | --- |
| CASP2L | Q-CASP2L-forward | CAAACTGTGAAACGAGAG |
|  | Q-CASP2L-reverse | CCAGGATGGAAGTAAATC |
|  | T7-CASP2L-forward | T7-TTACTCAACTTGTCGATGCACTC |
|  | T7-CASP2L-reverse | T7-GCTCCATTCTCCCACACTCTTAC |
| CASP8L | Q-CASP8L-forward | GTGGTTGATGGAAACATG |
|  | Q-CASP8L-reverse | ATAAGTGTCGTAGGGCAA |
|  | T7-CASP8L-forward | T7-CCCAGAGAATGTTTCAAGAGGTA |
|  | T7-CASP8L-reverse | T7-GAAAGAACACACAGGACCAGAAC |
| IAP | Q-IAP-forward | GGAAGAAACCAAAAGCAG |
|  | Q-IAP-reverse | TCATCCGTGTAGCAGATC |
|  | T7-IAP-forward | T7-GGGAACGGTTGAAGAGTTTTGAA |
|  | T7-IAP-reverse | T7-CACAGTGGAAGCATATCGTCTGG |
| Actin | Q-actin-forward | GCATTGGACTTTGAGCAGGA |
|  | Q-actin –reverse | AGGAACGATGGCTGGAAGAG |
| RGDV P8 | Q-G8-forward | GGGCTCTGTCTACACCCTTGAT |
|  | Q-G8-reverse | TCATAAGCCTTCAGAACTCCAA |
| GFP | T7-GFP-forward | T7-ATGTGCTGCAACATGAGCTCAC |
|  | T7-GFP-reverse | T7-TTACGCAAAGTACATGACTTTCTTG |
| VDAC | Q-VDAC-forward | GTTCTCTACAGGTGGTGTCTCA |
|  | Q-VDAC-reverse | ATCAGTATTCCATTTTTCCGTG |
|  | T7-VDAC-forward | T7-GGTTTCCTTGGACTCTTCGTTT |
|  | T7-VDAC-reverse | T7-TTCATTGAAGTTCTTGCCATCA |
| P8 | NB-P8-forward | TTGATTCAAGGGGCACAGAACGT |
|  | NB-P8-reverse | TTACCGCCTGATTAGCTGGCATA |
| Pns12 | NB-Pns12-forward | ATGACGAGCAACGAGGAAAACCC |
|  | NB-Pns12-reverse | CTCTGAGCGACTTCTCCACCTTT |

T7 sequence: ATTCTCTAGAAGCTTAATACGACTCACTATAGGG.

T7 denotes primers used for RNA silencing.

Q denotes primers used for RT-qPCR.

NB denotes primers used for Northern blots.
